# Synergistic Bioactive Ointment: ZnO Nanoparticles Combined with *Carica papaya* Latex and *Aloe Vera* Gel for Broad-Spectrum Biomedical Applications

**DOI:** 10.1371/journal.pone.0353765

**Published:** 2026-07-21

**Authors:** Geethma Ekanayake, Amavin Mendis, Supuni Wijayawardana, Charitha Thambiliyagodage, Madara Jayanetti

**Affiliations:** Department of Applied Sciences, Faculty of Humanities and Sciences, Sri Lanka Institute of Information Technology, Malabe, Sri Lanka; Amity University Noida, INDIA

## Abstract

The development of multifunctional topical formulations that combine natural bioactives with therapeutic nanomaterials offers a promising route to improved wound healing and management. *Carica papaya* fruit latex powder and *Aloe barbadensis Miller* gel were incorporated as active ingredients into an ointment base consisting of ZnO nanoparticles, cassava starch, and white petroleum jelly to formulate a novel topical medication with enhanced biological properties. According to the 2,2-diphenyl-1-picrylhydrazyl antioxidant assay, IC_50_ values of *Carica papaya* latex, *Aloe* gel, and ZnO nanoparticles were 454.54, 312.50, and 490.82 μg/mL, respectively. *Carica papaya* latex powder showed a protease activity of 58.40 units/mg of the solid. The ointment base exhibited the lowest blood clotting index (3.57 ± 6.09%), suggesting strong coagulation potential, whereas ZnO showed the highest (22.26 ± 1.09%), indicating minimal clotting ability under the current experimental setup. Erythrocyte adsorption was also highest in the ointment base (84.10 ± 1.09%), reinforcing its strong interaction with blood components. Cassava starch and petroleum jelly displayed moderate Red Blood Cell (RBC) attachment percentages. Platelet adhesion with the ointment base (61.55 ± 1.09%) and cassava starch (53.97 ± 2.40%) showed better platelet interaction than ZnO (29.1 ± 4.08%). Hemolysis data indicated that petroleum jelly caused the highest RBC lysis at all concentrations, while the ointment base showed the lowest activity, indicating better hemocompatibility. Clotting time analysis further highlighted the ointment base and petroleum jelly as effective pro-coagulants (13.30 s and 13.34 s, respectively), whereas ZnO (18.34 min) and cassava starch (48.34 s) exhibited delayed clotting. The ointment in which the base: active ingredient ratio is 7:3, and within, *Carica papaya* latex: *Aloe gel* ratio 1:4, shows the maximum antibacterial activity against *Staphylococcus aureus* and *Escherichia coli*. The MBC/MIC ratios of the above ointment for the two organisms were lower than 4, suggesting a bactericidal effect. The time-kill curves show a gradual reduction in bacterial survival over time, with the most significant reduction observed in the ointment with the highest proportion of *Aloe vera.*

## 1. Introduction

Wound healing is a vital and complex dynamic physiological process that restores the integrity of damaged tissues through a series of highly regulated biological events involving multiple overlapping phases and cell types. These include hemostasis, inflammation, proliferation, and remodeling [1]. During the hemostasis phase, platelets aggregate and form a fibrin clot, which stops bleeding and initiates cellular signaling through the release of growth factors such as PDGF (Platelet-derived growth factor) and TGF-β (Transforming Growth Factor beta) [2]. This sets the stage for the inflammatory phase, where neutrophils and macrophages infiltrate the wound to clear debris, neutralize pathogens, and secrete cytokines that modulate repair [3]. The antibacterial and antioxidant properties of biomaterials play a pivotal role in accelerating and improving wound healing outcomes. Antibacterial activity prevents microbial colonization and infection at the wound site, which is critical to avoiding delayed healing and chronic inflammation. Meanwhile, antioxidant capacity mitigates oxidative stress by neutralizing excessive reactive oxygen species (ROS) generated during the inflammatory phase, thus protecting cellular components and promoting tissue regeneration. Together, these dual functions maintain a balanced wound microenvironment, reduce the risk of infection-related complications, and enhance the proliferation and migration of fibroblasts and keratinocytes, ultimately leading to faster and more effective tissue repair. Proteolytic activity is a key factor in promoting efficient wound healing by aiding in the removal of necrotic tissue and facilitating the natural debridement process. By breaking down denatured proteins and damaged extracellular matrix components, proteolytic enzymes create a cleaner wound bed that supports cell migration, angiogenesis, and granulation tissue formation. This enzymatic action not only accelerates the transition from the inflammatory to the proliferative phase but also enhances nutrient and oxygen diffusion to regenerating tissues. When properly regulated, proteolytic activity contributes to faster re-epithelialization and improved overall healing outcomes [4–6].

Conventional wound care systems have historically focused on preventing infection, maintaining moisture balance, and protecting the wound site, rather than actively promoting wound regeneration. Examples include topical antiseptics such as povidone-iodine, silver sulfadiazine ointments for burn wounds, and antibiotic ointments like mupirocin and neomycin that are used for superficial infections [7]. Occlusive dressings, such as petroleum jelly based formulations and hydrocolloid patches, help preserve moisture and protect against microbial invasion while providing a physical barrier to further injury [8]. Traditional wound care treatments, including antibiotics, antiseptics, and synthetic ointments, are often limited by poor bioavailability, antimicrobial resistance, and a lack of regenerative support [9]. The overuse of antibiotics has fueled the rise of multidrug-resistant strains, reduced therapeutic efficacy, and increased treatment complexity [10]. Scarring after wounds, driven by excessive collagen deposition and aberrant remodeling, limits functional recovery and aesthetic outcomes [11]. Consequently, there is a growing need for formulations that not only fight infection but also stimulate tissue repair and reduce fibrosis [12]. To overcome the limitations of traditional systems, significant research has been directed toward advanced wound healing platforms that combine antimicrobial protection with regenerative support. Hydrogels, for example, provide a moist wound environment while acting as carriers for growth factors, stem cells, or nanoparticles. Silver-impregnated hydrogels have been shown to enhance antimicrobial protection with sustained release profiles [13]. Electrospun nanofiber scaffolds mimic the extracellular matrix (ECM), allowing for improved cell attachment and drug incorporation, while bioengineered skin substitutes, such as bilayered dermal equivalents or keratinocyte sheets, provide structural and biological cues to accelerate closure [14]. Nanotechnology-based interventions, including zinc oxide (ZnO), titanium dioxide, and silver nanoparticles, are increasingly integrated into ointments, dressings, and scaffolds for their antimicrobial and immunomodulatory effects [15,16]. Thus, research on formulations with enhanced antimicrobial activity along with protein debridement properties is crucial to develop effective wound care treatments [17,18]. Natural products have emerged as promising candidates in this aspect due to their biocompatibility, multifunctionality, and relatively lower toxicity [19,20]. Among novel strategies, increasing attention has been given to natural product-based systems and multifunctional nanoparticles, which directly relate to the components of the present study. The gel matrix of *Aloe barbadensis Miller* (*Aloe vera*) contains polysaccharides like acemannan, glycoproteins such as alprogen, and antioxidants including vitamins C and E, all of which contribute to macrophage activation, growth factor secretion, and angiogenesis enhancement, pro-inflammatory cytokines inhibition, and antimicrobial activity [21,22]. *Aloe vera* has been formulated into hydrogel dressings, hydrocolloid patches, and nanofiber mats, leveraging its polysaccharide (acemannan) and antioxidant content to promote angiogenesis, collagen deposition, and epithelialization [23,24]. A homogeneous and smooth morphology can be obtained in the formulations with *Aloe vera* gel as previous studies with amorphous, smooth films reported by Kaur et al. for *Aloe vera*/chitosan composites. *Carica papaya* fruit latex is rich in proteolytic enzymes such as papain, chymopapain, and caricain, which possess enzymatic debridement activity, enabling the removal of necrotic tissue while sparing healthy cells [25]. *Carica papaya* latex has been incorporated into ointments and debriding formulations to selectively remove necrotic tissue while enhancing fibroblast proliferation and granulation tissue formation [26]. Zinc oxide nanoparticles (ZnO NPs) have gained attention in medical applications and have been developed into topical gels, hydrogels, and nanofiber scaffolds due to their broad-spectrum antimicrobial activity, anti-inflammatory effects, and ability to stimulate cellular proliferation and angiogenesis [27,28]. ZnO NPs have gained considerable attention in pharmaceutical and biomedical applications due to their excellent antibacterial activity against a wide range of bacterial strains and their low toxicity to human cells at therapeutic concentrations. The antimicrobial mechanism of ZnO NPs involves the generation of reactive oxygen species (ROS), disruption of bacterial cell walls, and subsequent oxidative stress-induced cell death. These unique physicochemical properties make ZnO NPs a promising candidate for topical wound healing formulations [16,18,29,30].

Studies have shown the biocompatibility of ZnO NPs and their capacity to positively influence biological processes at the cellular level, supporting their safe and effective use in topical applications [28]. Despite their promise, these agents are rarely studied in combination, and most formulations face limitations related to stability, short shelf life, or lack of controlled release. Therefore, integrating these individual components within a stable excipient matrix represents a rational step toward a multifunctional, synergistic potential wound healing system that addresses both infection control and tissue regeneration. To ensure sustained drug release and formulation compatibility, white petroleum jelly and cassava (*Manihot esculenta Crantz*) starch were chosen as excipients. White petroleum jelly forms a hydrophobic barrier that prevents moisture loss, reduces microbial invasion, and maintains a moist wound environment conducive to wound healing [31] while cassava starch, a biocompatible polysaccharide, contributes to improved absorbency. It also controls the release of active compounds, making it suitable for wound dressings [32]. Studies have shown that the incorporation of natural biopolymers enhance the structural integrity, functional properties, and biocompatibility of composite formulations [20]

In the present study, we formulated a novel ointment composed of *Carica papaya* latex powder, *Aloe barbadensis Miller* gel, and ZnO nanoparticles, embedded within a base of white petroleum jelly and cassava starch. The formulation was characterized in vitro by its physicochemical properties, hemocompatibility, protease activity, antioxidant potential, and antimicrobial efficacy, which are important parameters of an efficient wound care system. Although the therapeutic potential was not evaluated under an in vivo setup, the study aims to provide valuable insights required to advance this formulation toward clinical application. It was aimed to evaluate the synergistic therapeutic effects of this multi-component system and establish its potential as a bioactive topical platform for enhanced wound management.

## 2. Methodology

### 2.1. Chemicals and materials

Fresh papaya latex was obtained from, raw, unripe *Carica papaya* L. (cv. Red Lady), Fresh *Aloe vera* gel was obtained from *Aloe barbadensis Miller* (syn. *Aloe vera* (L.) Burm.f.), both locally grown at Horticultural Crop Research and Development Institute, (HORDI), Gannoruwa, Peradeniya, Sri Lanka. Potassium metabisulfite (KMS) K₂S₂O₅ (≥ 98%), DPPH (2,2-Diphenyl-1-picrylhydrazyl) (>95%), Sodium acetate (CH₃COONa) (≥99%), Casein, Hi-LR™ (92%), Sodium Carbonate (Na₂CO₃) (99%), Mueller Hinton Agar (MHA), and Luria Bertani Broth (LB broth) were purchased from HiMedia Laboratories (Germany), Sodium hypochlorite (NaOCl) (5%) was procured from Atom Scientific Limited (Hyde, Greater Manchester, England), Zinc acetate dehydrate (Zn(CH₃CO₂)₂·2H₂O) (≥ 99.5%), NaOH pellets ((≥ 98%), Calcium acetate (Ca(C₂H₃O₂)₂) (≥ 99%), Trichloroacetic acid (TCA) (C₂HCl₃O₂) (99%), L-Tyrosine (C₉H₁₁NO₃) (99%), Folin & Ciocalteu Phenol (FCP) Reagent AR (~2.0 N), Dimethyl sulfoxide (DMSO) (99%), and Acetonitrile (ACN) (CH_3_CN) were purchased from Sisco Research Laboratories (Pvt) Ltd. (Mumbai, India). Methanol (CH₃OH) (≥ 99.6%), and Acetic acid (CH_3_COOH) (≥99%) were procured from Sigma Aldrich Co Ltd (Gillingham SP8 4XT, United Kingdom). All of the chemicals utilized in the experiments were of analytical grade and were used without further purification.

Bacterial pathogens used for the study, the test organisms, Gram-negative *Escherichia coli*, and Gram-positive *Staphylococcus aureus*, were procured from the Medical Research Institute, Sri Lanka.

### 2.2. Study design and period

This experimental study was carried out between 12/11/2024 and 14/03/2025. During this time, all sample collection, formulation development, and *in-vitro* testing were carried out in the manner outlined below. Sample preparation was completed between 12/11/2024 and 02/12/2024, whereas the *in-vitro* DPPH assay and the protease activity determination assay were conducted between 14/12/2024 and 31/12/2024. Antimicrobial activity of the synthesized materials was assessed from 02/01/2025 to 12/01/2025. Since freshly made formulations were used for each trial set, whole blood was obtained at various times between 13/02/2025 and 10/03/2025, in order to ensure sample-to-assay integrity.

### 2.3. Isolation of latex from *Carica papaya* fruits

Fresh papaya latex was collected from locally grown raw, unripe *Carica papaya* fruits from the Horticultural Crop Research and Development Institute (HORDI), Sri Lanka. The latex collection was done between 6:00 am and 10:00 am. Initially, four to six longitudinal incisions were made on the unripe fruit using a sharp stainless-steel knife. The exuded latex was allowed to run down the fruit and drip into collecting devices. Collected latex was mixed with potassium metabisulfite (KMS) (K_2_S_2_O_5_) in a ratio of 0.5% W/W. This treatment is a standard practice to stabilize papain, as KMS serves as a mild reducing agent to maintain the enzyme’s catalytically essential thiol group in its active state and prevent oxidative degradation during processing [33]

### 2.4. Drying and storage of *Carica papaya* fruit latex

The collected latex was dried using a hot air oven at 40 °C for 2 days until all the moisture was gone. After drying, the latex appeared to be a lightly sticky solid substance on the tray. The solid particles were then ground into a fine powder, transferred to an opaque polypropylene airtight container, and stored at −20 °C until used for analysis [33,34].

### 2.5. Preparation of *Aloe barbadensis Miller* gel

Fresh *Aloe barbadensis Miller* leaves obtained were washed, surface-sterilized with mild chlorine, and cut into pieces. The thick epidermis was removed, and the inner parenchymal gel was scooped out, minced, and homogenized into a smooth paste. The prepared gel was then stored at 4 °C for later use [35].

### 2.6. Synthesis of ZnO nanoparticles

A 1 M zinc acetate (Zn(CH₃CO₂)₂·2H₂O) solution and a 2 M NaOH solution (of equal volumes) were prepared. The NaOH solution was then added drop wise to the zinc acetate solution under continuous stirring on a magnetic stirrer at room temperature (25 °C). The reaction mixture was stirred overnight to ensure complete precipitation of ZnO. The resulting precipitate was collected and washed repeatedly with deionized water until the supernatant reached a neutral pH and all residual acetate ions were removed. Finally, the purified ZnO precipitate was dried in an oven at 80 °C to obtain ZnO nanoparticles [36].

### 2.7. Preparation of the topical medication ointment

The ointment base was prepared with slight modifications to the Zinc Oxide Ointment formula (USP) by combining white petroleum jelly, ZnO nanoparticles, and cassava (*Manihot esculenta Crantz*) starch in a 2:1:1 (w/w) ratio. To determine the optimal synergistic ratio between the active ingredients, *Aloe barbadensis Miller* gel and *Carica papaya* fruit latex powder were then incorporated into the base at a fixed ratio of 7:3 (base: actives). To evaluate compositional effects, the *Carica papaya* latex-to-*Aloe vera* ratio was varied (1:1, 1:2, 2:1, 1:4, and 4:1) while maintaining active ingredients at 30% of the total formulation. Additionally, formulations with varying base-to-active ratios (9:1, 8:2, 7:3, 6:4, and 5:5) were also prepared to study the optimal total concentration of active ingredients, maintaining a constant *Carica papaya* latex-to-*Aloe* *vera* ratio of 1:4. Each mixture was thoroughly mixed and homogenized to ensure uniformity and stored in airtight containers under ambient conditions for further analysis [35,37,38]

### 2.8. Characterization

The changes in the surface chemical bonding and surface composition were characterized by using an ATR device employing Fourier Transform Infrared (FT-IR) spectroscopy (Nicolet Avatar series 330) (Thermo Fisher Scientific, Madison, WI, USA), ranging from 400 to 4000 cm^-^1. XRD analysis was carried out on an X-ray diffractometer (X’Pert-PRO) (Malvern Panalytical, Almelo, The Netherlands). The high-resolution XRD patterns were measured at 3 KW with Cu target using a scintillation counter (λ = 0.15406 nm) at 40 kV and 40 mA, and were recorded in the range of diffraction angle (2θ) 3° to 80° at 2°/min scanning rate. The surface morphology of the synthesized nanoparticles was characterized using a ZEISS EVO 18 RESEARCH instrument (Carl Zeiss AG, Oberkochen, Germany) [39].

#### 2.8.1. Fourier transform infrared (FTIR) spectroscopy.

FTIR spectroscopy was performed using a Nicolet Avatar series 330 spectrometer (Thermo Fisher Scientific, Madison, WI, USA) equipped with an ATR accessory to identify functional groups in the ointment formulations as well as individual components. A small portion of each sample was placed directly onto the ATR crystal, ensuring complete surface coverage. The spectra were recorded over the wavenumber range of 4000–400 cm ⁻ ¹, with 32 scans co-added at a resolution of 4 cm ⁻ ¹ using the Omnic software. The ATR crystal was cleaned with ethanol and a background spectrum was collected before each sample run to minimize environmental interferences [40]

#### 2.8.2. X-ray diffraction (XRD) analysis.

XRD analysis was conducted using an X’Pert-PRO diffractometer (Malvern Panalytical, Almelo, The Netherlands) to examine the crystalline nature and phase purity of the samples. The sample was ground to a fine powder and packed into a flat sample holder to ensure a smooth, level surface. Diffraction patterns were recorded in the Bragg-Brentano geometry using Cu Kα radiation (λ = 0.15406 nm) generated at 40 kV and 40 mA, with a scan range of 2θ = 3°–80° at a scanning rate of 2°/min. The resulting diffractograms were analyzed using HighScore Plus software, and phase identification was performed by comparison with ICDD PDF database reference patterns [41]

#### 2.8.3. Scanning electron microscopy (SEM) analysis.

Surface morphology of the components were examined using a ZEISS EVO 18 RESEARCH scanning electron microscope (Carl Zeiss AG, Oberkochen, Germany). The powders were dispersed in ethanol, and a drop of the suspension was placed onto a clean aluminum stub and allowed to air-dry. To prevent charging during imaging, the sample was sputter-coated with a thin gold layer using a sputter coater. The micrographs were captured at an accelerating voltage of 20 kV and a working distance of approximately 10 mm at various magnifications (up to 50,000×) using the secondary electron detector and SmartSEM software [42]

### 2.9. HPLC analysis

The *Aloe barbadensis Miller* gel and the *Carica papaya* latex methanolic extract were filtered through a 0.45 μm nylon syringe and directly injected for analysis. The HPLC fingerprint of the methanolic extracts was obtained using an Agilent 1200 HPLC system equipped with a ZORBAX Eclipse XDB-C18 column (4.6 mm × 150 mm, 5 micron). Detection was carried out at 254 nm using a UV–vis detector for the *Aloe barbadensis Miller* gel. The mobile phase consisted of (A) acetonitrile (HPLC grade) and (B) buffer (Water: Acetonitrile: Acetic Acid (89.9: 10: 0.1, v/v)) delivered at a flow rate of 1.0 mL/min. Gradient elution was employed as follows: initially an isocratic condition with 5% A (95% B) from 5–10 min, linearly increased to 15% (A) at 15 min and held isocratic for 10 min, after which it was linearly increased to 22% A over 30 min (25–55 min), followed by re-equilibration to initial conditions by over 2 min (55–57 min). The total running time was 57 min, and the column temperature was maintained at 45 °C. The injection volume was 20.0 μL. The mobile phase for the *Carica papaya* latex extract consisted of (A) acetonitrile (HPLC grade) and (B) acetic acid (0.5%), which was delivered at 1 mL/min. The elution program was initiated at 0% A (100% B), linearly increased to 30% (A) at 40 min, 60% (A) at 60 min, and 90% (A) at 62 min, held until 68 min, followed by re-equilibration to initial conditions by 70 min. The total running time was 70 min, and the column temperature was maintained at 30 °C. The injection volume was 20.0 μL, and the detection was carried out at 280 nm using a UV–vis detector [43]

### 2.10. Antioxidant capacity assay – DPPH assay

According to an optimized protocol [44], utilizing a stable 2,2-diphenyl-1-picrylhydrazyl (DPPH) solution, the antioxidant potential of *Carica papaya* fruit latex, *Aloe barbadensis miller* gel, and ZnO nanoparticles was assessed. To the prepared concentration series (100, 200,300,400,500,600,700 μg/mL) of the extracts, 0.5 mL of the 1 mM DPPH stock solution was added, and the absorbance was measured against the blank solution at 517 nm after 30-minute incubation in the dark. All experiments were performed in triplicate (n = 3), and the results were expressed as mean % inhibition of the DPPH radical (RSA%) ± standard error (SE), calculated using the following formula:


$$\begin{array}{c} \%\text{ inhibition}/\text{ RSA}\%\;=\;\frac{Abs\;of\;control-Abs\;of\;sample}{Abs\;of\;comtrol}\;\times \text{1}\text{0}\text{0} \end{array}$$

The IC₅₀ value, representing the concentration of the sample required to scavenge 50% of the DPPH radicals, was determined by plotting RSA% against concentration using a linear regression model.

### 2.11. Whole blood assays

This study was carried out following the relevant national and institutional guidelines for research involving human samples. The protocol for obtaining and using human blood samples for in vitro assays was reviewed and approved by the Ethics Review Committee of the Faculty of Humanities and Sciences, Sri Lanka Institute of Information Technology (Approved Date: 11 February 2025). Written informed consent was obtained from all participants before blood sample collection.

Whole blood coagulation (Blood clotting index) [45], Red blood cell (RBC) attachment, Platelet adhesion, Hemolysis, and clotting time assays [46] were performed for the samples, and the detailed protocols are available in the supplementary information.

### 2.12. Protease activity determination assay for *Carica papaya* latex

An enzyme diluent solution, which consists of 10 mM Sodium Acetate Buffer with 5 mM Calcium Acetate, pH 7.5, at 37 °C, was prepared, and the *Carica papaya* latex powder was dissolved in it. To each test vial, 5 mL of 0.65% casein solution was added, followed by equilibration in a 37 °C water bath for 5 minutes. Varying volumes of the enzyme solution were then added to the three test vials (excluding the blank), mixed by swirling, and incubated at 37 °C for exactly 10 minutes to allow tyrosine liberation via proteolysis. After incubation, 5 mL of trichloroacetic acid (TCA) reagent was added to each vial to terminate the reaction. The enzyme solution was then adjusted to a final volume of 1 mL in all vials (including the blank) to standardize volumes and account for enzyme-specific absorbance. The solutions were incubated for an additional 30 minutes at 37°C. Concurrently, tyrosine standard dilutions were prepared in six vials using 1.1 mM tyrosine stock (0.05–0.50 mL) and diluted to 2 mL with purified water (no tyrosine added to the blank). Post-incubation, all test solutions and blanks were filtered through 0.45 µm polyether sulfone syringe filters to remove insoluble material. Filtrates (2 mL) were then transferred to vials, followed by sequential addition of 5 mL sodium carbonate and 1 mL Folin’s reagent to all standards and test samples. After mixing and a final 30-minute incubation at 37 °C, the developed color (proportional to tyrosine concentration) was quantified spectrophotometrically at 660 nm.

A standard curve was generated by plotting the absorbance against tyrosine concentration, and its slope equation was used to determine tyrosine liberation in test samples (corrected for blank absorbance). Protease activity (units/mL) was calculated as:


$$\begin{array}{c} \text{Units}/\text{mL enzyme }=\;((\mu \text{M tyrosine})\times \text{11})/\;(\text{1}\;*\text{ 10 }*\;\text{2}) \end{array}$$

where 11 = total assay volume (mL), 10 = reaction time (minutes), 1 = volume of enzyme used (mL), and 3 = volume used for colorimetry (mL). To express activity per solid mass (units/mg), the units/mL value was divided by the original solid protease concentration (mg/mL) [47].

### 2.13. Antimicrobial assays

#### 2.13.1. Microbial strain and inoculum preparation.

*Escherichia coli* (Gram-negative) and *Staphylococcus aureus* (Gram-positive), common wound-infecting bacteria, were obtained from the Medical Research Institute, Sri Lanka, and maintained on agar slants at 4 °C. For antibacterial testing, 24-hour-old cultures grown in Luria Bertani broth were standardized to 5 × 10^5^ CFU/mL using the 0.5 McFarland standard and a UV 1900 Shimadzu UV Visible Double Beam Spectrophotometer, (Shimadzu Corporation, Kyoto, Japan).

#### 2.13.2. Agar well diffusion method.

Mueller Hinton Agar plates were inoculated with 5 × 10^5 CFU/mL bacterial suspensions, and wells were filled with 70 μL of test solutions at 10, 20, or 40 mg/mL. Gentamicin and DMSO served as positive and negative controls, respectively. Each test was performed in triplicates, and plates were incubated at 37 °C for 18 hours before measuring zones of inhibition [48].

#### 2.13.3. Minimum inhibitory concentration (MIC) and minimum bactericidal concentration (MBC).

Antibacterial solutions were prepared at concentrations ranging from 0.625 to 80 mg/mL, with 0 mg/mL as the control. For MIC determination, equal volumes of standardized microbial cultures and test solutions in LB broth were incubated, and the lowest concentration showing no turbidity was recorded as the MIC. For MBC, aliquots from these cultures were plated on Mueller-Hinton agar, and the lowest concentration that completely inhibited bacterial growth, corresponding to a 99.9% reduction, was identified as the MBC. Both MIC and MBC assays were incubated at 37 °C for 18–24 hours (48).

#### 2.13.4. Time-kill synergy assay.

The time-kill synergy assay was conducted using the broth macro dilution method with an inoculum of ~5 × 10^5^ CFU/mL. Test samples were mixed with LB broth in a 2:1 ratio, incubated at 37 °C with shaking, and bacterial growth was monitored by measuring optical density at 600 nm every hour for 12 hours. Growth curves were plotted to evaluate antibacterial activity, with gentamicin as the positive control and DMSO as the negative control [48].

S1 Scheme in the supplementary information section depicts the overall experimental design of the study.

### 2.14. Statistical analysis

All the experiments were triplicated, and the data from each experiment were statistically analyzed using SPSS Statistics (Version 27, SPSS Inc., Chicago, Illinois, USA), and the results were reported as mean ± standard error (SE). The confidence level of 95% was set as Statistical significance. One-way analysis of variance (ANOVA) was used to determine the differences among the treatment means.

## 3. Results and discussion

### 3.1. SEM analysis

**Fig 1:**
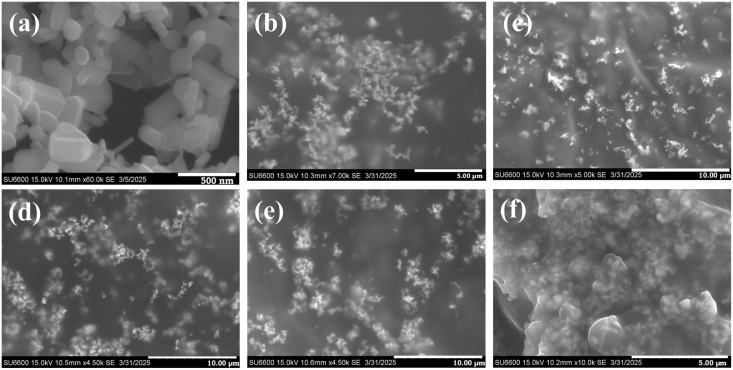
SEM image of (a) ZnO nanoparticles (b) ointment base (c) ointment (base:active 9:1, *Carica papaya* latex:*Aloe* 1:4) (d) ointment (base:active 7:3, *Carica papaya* latex:*Aloe* 1:2) (e) ointment (base:active 7:3, *Carica papaya* latex:*Aloe* 1:1) (f) ointment (base:active 7:3, *Carica papaya* latex:*Aloe* 4:1).

The SEM image of the ZnO nanoparticles (Fig 1 (a)) reveals a notably heterogeneous morphology comprising spherical, rod-like, and irregularly shaped particles, indicating non-uniform growth dynamics during synthesis. The coexistence of different morphologies suggests multiple nucleation and crystal growth pathways were active, possibly influenced by variations in local super saturation, temperature gradients, or precursor concentration. The presence of rod-shaped structures is characteristic of anisotropic crystal growth. Spherical particles, on the other hand, are indicative of isotropic growth conditions where nucleation is rapid, and growth is uniformly distributed in all directions. Irregular particles may result from the coalescence of primary particles, incomplete crystallization, or secondary agglomeration during drying or sample preparation [7,49,50]. The mixed morphology can significantly affect the physical and chemical properties of the ZnO nanoparticles, including surface area, reactivity, and potential application-specific interactions (7).

Fig 1 (b) shows the SEM micrograph of the formulated ointment base containing ZnO nanoparticles, cassava starch, and petroleum jelly that reveals a heterogeneous microstructure characterized by clustered, granular particles and regions of diffuse contrast. The brighter, agglomerated zones are indicative of ZnO nanoparticles, which tend to cluster due to their high surface energy, especially within semi-solid matrices like petroleum jelly. The SEM micrograph of the ointment formulated with a 90:10 base-to-active ratio (base: ZnO nanoparticles, cassava starch, petroleum jelly; active: *Carica papaya* latex: *Aloe vera* at 1:4) (Fig 1 (c)) shows a markedly more heterogeneous and textured surface compared to the base formulation alone. While distinct spherical ZnO nanoparticles are no longer clearly visible, this may be due to partial encapsulation, embedded in the ointment base or surface coverage by *Aloe vera* and papain components, as well as agglomeration into larger, indistinct clusters. This appearance was observed in other SEM images (Figs 1 (d), (e), and (f)) corresponding to different formulas ointment (base:active 7:3, *Carica papaya* latex:*Aloe* 1:2), ointment (base:active 7:3, *Carica papaya* latex:*Alo*e 1:1), ointment (base:active 7:3, *Carica papaya* latex:*Aloe* 4:1), respectively.

The SEM micrograph of the ointment formulation with a 70:30 base-to-active ratio and a 4:1 *Carica papaya* latex to *Aloe vera* distribution (Figs 1 (f)) reveals significant microstructural disruption compared to lower active-loaded systems (e.g., 9:1 ratio). The petroleum jelly–cassava starch–ZnO nanoparticle (ZnO NP) base remains discernible but exhibits a fragmented, less cohesive morphology, suggesting that the elevated active concentration compromises the structural integrity of the gel-starch matrix. ZnO NPs are not distinguishable as discrete entities, likely due to agglomeration or their encapsulation within the disrupted polymeric network. The overall microstructure is dominated by dense, bright, irregularly shaped aggregates, consistent with papain-rich domains, which are scattered unevenly throughout the matrix. These aggregates contribute to a highly granular, heterogeneous surface topography, markedly lacking the smoother appearance observed in formulations with higher *Aloe vera* content (e.g., *Carica papaya*: *Aloe* 1:2), which are known for their homogenizing and film-forming properties. The limited *Aloe* content in this formulation fails to adequately modulate the roughness induced by the abundant papain, resulting in prominent clusters and evident phase separation. These papain-dense zones contrast sharply with the surrounding base, indicating poor miscibility and reinforcing the lack of uniform dispersion within the system. Overall, the SEM findings underscore the challenges of maintaining matrix cohesion and nanoparticle stability at higher active loadings, particularly when papain dominates the bioactive phase.

The observed microstructural disruption and phase separation at high papain loadings are consistent with recent reports where protein-rich botanical extracts formulated into semi-solid matrices exhibited poor miscibility and aggregation. Kaur et al. demonstrated that *Aloe vera* gel enhances matrix cohesion and reduces surface heterogeneity through its film-forming properties, supporting our SEM observation that higher *Aloe* content (e.g., 1:2 ratio) yielded smoother, more homogeneous surfaces compared to papain-dominated formulations [22,51,52].

### 3.2. XRD analysis

**Fig 2:**
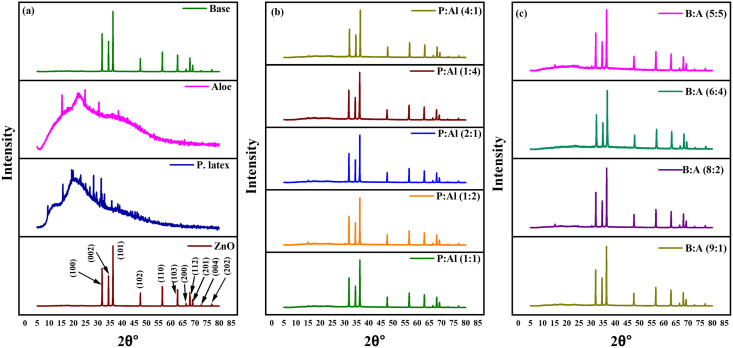
XRD patterns of the (a) individual components (b) and (c) ointment formulations.

The X-ray diffraction (XRD) analysis provided detailed insight into the structural characteristics, crystallographic orientation, and phase composition of the individual components, ZnO nanoparticles, *Carica papaya* latex powder, *Aloe barbadensis Miller* gel, the ointment base, and the final formulations with varying ratios of bioactive ingredients. The XRD pattern of ZnO nanoparticles (Fig 2 (a)) exhibited sharp and intense diffraction peaks at 2θ values of 32.07°, 34.74°, 36.55°, 47.83°, 56.85°, 63.13°, 66.58°, 68.19°, 69.35°, 73.26° and 77.70°, corresponding to the (100), (002), (101), (102), (110), (103), (200), (112), (201), (004) and (202) crystal planes of the hexagonal wurtzite structure (JCPDS-36–1451). The calculated interplanar spacing (d) for the (101) reflection at 36.55° was 0.2456 nm, while the average crystallite size determined using the Debye–Scherrer equation was 48.50 nm. These results confirm the phase purity and high crystallinity of ZnO, with no secondary or impurity phases detected, demonstrating the effectiveness of the synthesis route.

In contrast, the *Carica papaya* latex and *Aloe barbadensis miller* gel displayed broad humps in their XRD patterns, indicative of their predominantly amorphous nature. The papaya latex showed a characteristic diffuse peak centered around 28.25° with a corresponding d-spacing of 0.32 nm, while *Aloe vera* exhibited a broader feature at 15.25° with a d-spacing of 0.58 nm. The lack of sharp reflections highlights the disordered molecular arrangement in these biopolymers, consistent with previous reports on natural biomaterials [53,54]. The ointment base exhibited diffraction peaks identical to those of ZnO, dominated by sharp reflections at 2θ = 32.07°, 34.74°, and 36.55°, confirming that the crystalline character is primarily derived from the ZnO incorporated within the base formulation. The high-intensity peaks further verify that the base ointment preserves the crystallinity of ZnO nanoparticles without introducing additional crystalline phases. For the composite formulations (Figs 2 (b) and (c)), systematic variations were observed with different ratios of *Carica papaya* latex and *Aloe vera*. In the P:A series with a fixed base:active ratio of 70:30, the XRD profiles demonstrated distinct differences depending on the relative proportions of *Carica papaya* and *Aloe*. In P:A (1:1), reflections were observed at 14.99°, 28.26°, and 36.33°, corresponding to d-spacings of 0.59, 0.32, and 0.25 nm, with crystallite sizes of 61.73, 77.96, and 77.58 nm, respectively. When *Aloe* content was increased (P:A 1:4), the ZnO peak at 36.26° remained prominent, but the calculated crystallite size reduced to 33.79 nm, suggesting structural disorder introduced by the organic matrix. Conversely, in papaya-rich formulations such as P:A (4:1), the major reflections appeared at 15.65°, 28.44°, and 36.52°, with crystallite sizes of 41.25, 77.96, and 77.57 nm, respectively, indicating partial recovery of ZnO crystallinity, likely due to stabilization effects of papaya latex.

In the B:A series, where P:A was fixed at 1:4 and the base:active ratio varied, changes in the diffraction profile reflected the influence of bioactive content. B:A (9:1) showed broad peaks at 15.19° and 36.36°, with the smallest crystallite size of 32.53 nm, indicating disruption of ZnO order due to excess base diluting the actives. As the ratio shifted to B:A (5:5), the diffraction peak at 36.41° became sharper, with a crystallite size of 77.57 nm, signifying improved crystalline order through effective incorporation of the bioactive matrix. Overall, the XRD results confirm that ZnO nanoparticles retain their crystalline nature in the formulations, while *Carica papaya* latex and *Aloe* contribute amorphous characteristics. The interaction between bioactive components and ZnO is strongly dependent on their relative proportions, influencing crystallite size, peak sharpness, and structural stability in the multifunctional wound healing formulations. Recent studies confirm that polymer-ZnO interactions reduce crystallinity. Jia et al. reported a decrease in ZnO crystallite size from 41.1 nm to 21.5 nm with increasing lentinan content [55], while Vyas et al. demonstrated reduced crystallinity in ZnO-embedded bioplastic films [56]. These findings align with our observation of reduced crystallite size (from 77.58 nm to 33.79 nm) with increasing *Aloe vera* content.

### 3.3. FTIR analysis

**Fig 3:**
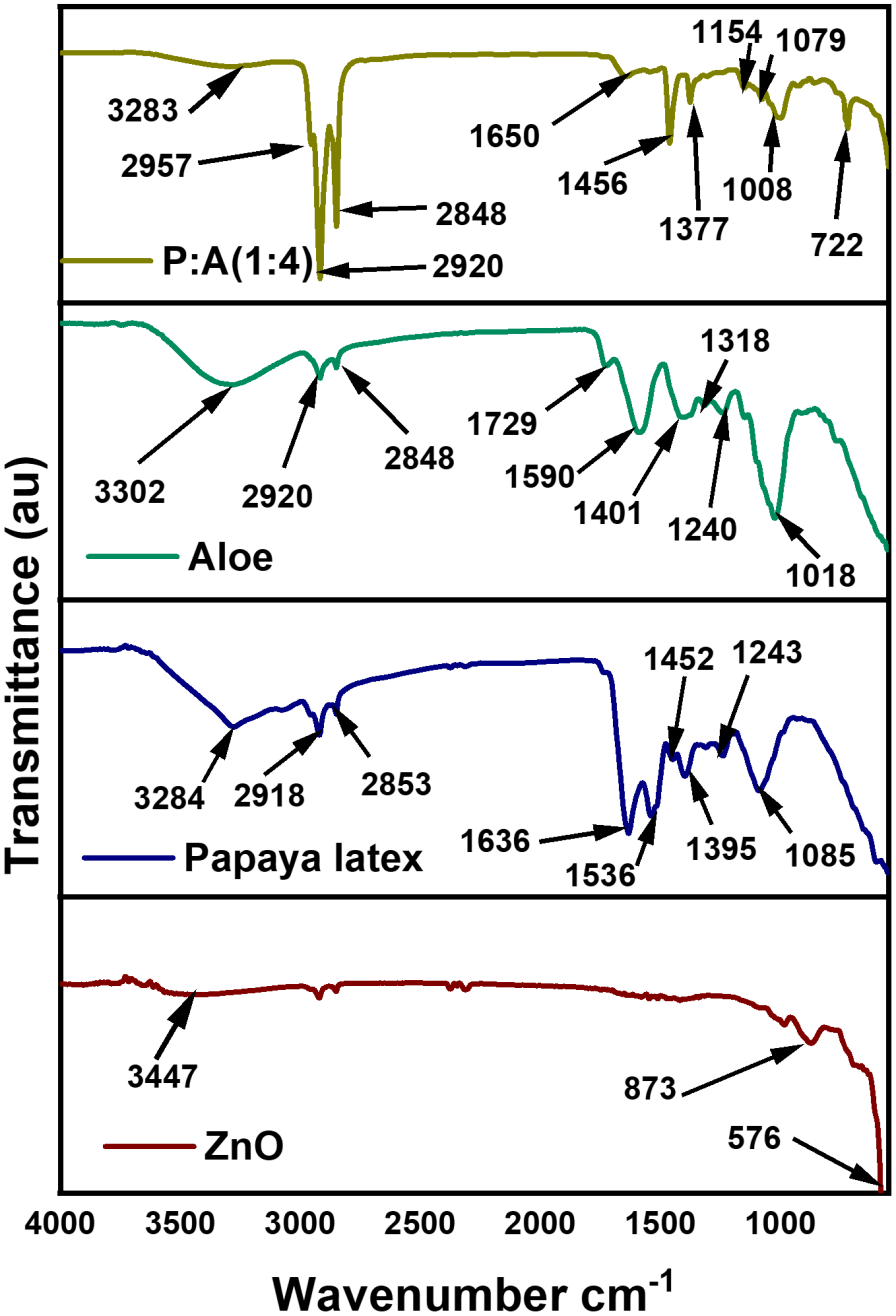
FT-IR spectra of the materials.

The Fourier Transform Infrared (FT-IR) spectra (Fig 3) provided definitive insights into the functional groups and molecular interactions of the formulation components, ZnO nanoparticles, *Carica papaya* latex, *Aloe barbadensis miller* gel, the ointment base, and the final ointments. At 576 cm ⁻ ¹, a strong Zn–O stretching vibration confirmed the crystalline integrity of ZnO. A weaker band at 873 cm ⁻ ¹, attributed to C = C out-of-plane bending, indicated trace surface interactions with unsaturated atmospheric species. The absence of organic absorptions verified the purity of ZnO. At the same time, the broad 3447 cm ⁻ ¹ O–H stretch reflected adsorbed hydroxyl groups and bound water, features that contribute to nanoparticle stability and reactivity.

The latex spectrum revealed rich biochemical signatures. The 1085 cm ⁻ ¹ C–O stretching band confirmed carbohydrate and glycosidic linkages, while 1395 cm ⁻ ¹ (–COOH bending) indicated carboxylic acids. Prominent amide II (1536 cm ⁻ ¹) and amide I (1636 cm ⁻ ¹) absorptions confirmed proteinaceous constituents, notably papain. The C–H stretches at 2853 and 2918 cm ⁻ ¹ reflected lipid residues, and the broad O–H stretch at 3284 cm ⁻ ¹ signified hydroxyl-rich polysaccharides, underscoring its enzymatic and antimicrobial bioactivity. *Aloe* gel exhibited strong C–O stretches (1018 cm ⁻ ¹), consistent with acemannan polysaccharides, alongside 1240 and 1401 cm ⁻ ¹ vibrations indicative of carbohydrate/lipid components. The 1590 cm ⁻ ¹ C = C band indicated unsaturated compounds, while the 1729 cm ⁻ ¹ C = O stretching confirmed carboxylic acids. Lipid-associated C–H stretching (2848 and 2920 cm ⁻ ¹) and a broad O–H stretch at 3302 cm ⁻ ¹ reflected the gel’s polysaccharide-rich, bioactive profile supporting anti-inflammatory and regenerative functions.

The ointment base exhibited C–O stretching (1008 cm ⁻ ¹) and amide I at 1650 cm ⁻ ¹, consistent with polysaccharide–protein compatibility. The final ointment spectrum integrated the features of all components. The 576 cm ⁻ ¹ Zn–O band persisted, verifying nanoparticle dispersion within the matrix. The 873 cm ⁻ ¹ (C = C bending) mode has broadened, reflecting the interaction between the unsaturated groups of *Carica papaya* and *Aloe*. Intact amide I and II bands (1650 and 1536 cm ⁻ ¹) confirmed papain stability, while the 3283 cm ⁻ ¹ O–H band broadened and shifted, indicating extensive hydrogen bonding among *Aloe vera*, *Carica papaya*, and the ointment base. Shifts in the C–H (2848–2957 cm ⁻ ¹) and C = O (~1650–1729 cm ⁻ ¹) bands suggested the presence of van der Waals forces and hydrogen-bonding interactions. Partial suppression of fingerprint-region peaks further supported molecular-level integration of ZnO within the organic base.

Collectively, the FT-IR spectra confirmed that all components retained their chemical integrity while establishing hydrogen bonding, van der Waals interactions, and nanoscale embedding of ZnO. The observed band shifts and broadenings demonstrate that the formulation is not a simple physical mixture but a synergistically integrated molecular system, ensuring both stability and therapeutic efficacy in wound healing. These spectral observations are consistent with recent findings on ZnO-biopolymer systems, where FTIR analysis confirmed hydrogen bonding and molecular-level integration between ZnO nanoparticles and organic matrices [57]. The O-H band broadening and Zn-O peak persistence observed in our study align with reports on *Aloe vera* mediated ZnO nanocomposites, which similarly demonstrated that bioactive phytochemicals facilitate strong interfacial interactions through hydrogen bonding and van der Waals forces [58]. These interactions are critical for ensuring formulation stability and therapeutic efficacy, as they prevent phase separation and maintain the structural integrity of the bioactive components [59].

### 3.4. HPLC analysis

The HPLC fingerprint of *Aloe barbadensis Miller* gel and papaya latex extract was analyzed to identify the presence of bioactive compounds present in each extract, as the biological activities are related to the relative composition of these compounds (S1 Fig). Chromatogram of the *Aloe* gel exhibited one major peak at 7.98 min, which corresponds to the aloesin, a potent tyrosinase inhibitor known for its wound healing properties. In the studies conducted by Bozzi et al., Dell’Agli et al., and Zhao et al [60–62] have reported aloesin elution at ~7.9 min, validating these results. This prominent peak aligns with the established retention behavior of aloesin, a key chromone marker documented in prior studies [63,64]. The chromatogram of the papaya latex consists of a few characteristic peaks, including a notable peak at around 5.19 minutes, presumably associated with carpaine, the principal alkaloid responsible for proteolytic and antibacterial properties [65]. Despite HPLC’s limitations for macromolecules like proteins, the presence of papain was independently confirmed using the protease activity assay, which measures enzymatic hydrolysis and directly confirms functional protein content.

### 3.5. Antioxidant activity – DPPH (2,2-diphenyl-1-picrylhydrazyl) assay

**Fig 4:**
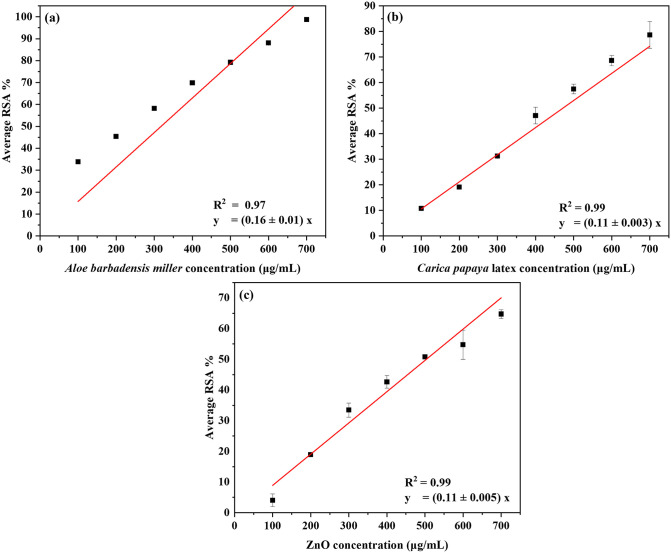
Antioxidant activity (DPPH assay) of (a) *Aloe barbadensis Miller* gel, (b) *Carica papaya* latex powder, (c) ZnO nanoparticles.

The DPPH radical scavenging assay results underscore the differential yet complementary antioxidant capacities of *Carica papaya* latex powder, *Aloe barbadensis Miller* (*Aloe vera*) gel, and Zinc oxide (ZnO) nanoparticles, each contributing to the neutralization of free radicals through distinct biochemical mechanisms. The dose-dependent increase in radical scavenging activity (RSA) observed for all samples validates their potential as oxidative stress modulators. Fig 4 and S2 Fig indicate the variation of the RSA% of the test sample over the tested concentration range. Among them, *Aloe barbadensis Miller* gel demonstrated the highest antioxidant efficacy (Fig 4(a)), with an RSA approaching 99% at 700 µg/mL and an IC₅₀ of 312.5 µg/mL, indicating its robust electron- and hydrogen-donating capacity. This activity is largely attributed to its rich phytochemical composition, encompassing vitamins C and E, flavonoids, polysaccharides (notably acemannan), and phenolic acids, which collectively function via hydrogen atom transfer (HAT) and single electron transfer (SET) mechanisms to stabilize reactive species such as DPPH^•^. Previous studies have shown that *Aloe vera* gel incorporation improved the rheological and antioxidant characteristics of biopolymer-based films [22]. The IC_50_ value for the sun-dried *Aloe Vera* extract was reported to be 2.95 ± 0.03 mg/mL in a study conducted by Hossen et al. [66], and Yahya et al. have reported a DPPH Scavenging (%) of 68.1% at 1000 μg/mL for *Aloe vera* gel extract [67]. However, some previous studies have reported a lower IC_50_ of 14.21 µg/mL for Aloe latex and 42 µg/mL for silver nanoparticles of Aloe Vera [68,69]. The differences in the results are due to the solvent systems used for the extraction and the phytochemical constituents of the different parts of the plant, as elucidated by Manye et al. [70]. *Carica papaya* latex also showed substantial antioxidant capacity (IC₅₀: 454.54 µg/mL) (Fig 4 (b)) mediated by phenolic compounds and proteolytic enzymes like papain, chymopapain, glycyl endopeptidase, and caricain. These enzymes, while primarily recognized for proteolytic action, indirectly support redox balance by modulating inflammatory cascades and facilitating cellular turnover. Papain, which accounts for the majority of the enzyme fraction of latex, has been studied for its antioxidant activity. The higher purity of the papain is responsible for the higher RSA% [71]. ZnO nanoparticles exhibited the lowest RSA (IC₅₀: 490.82 µg/mL) (Fig 4 (c)), likely due to their inorganic nature and less direct participation in HAT or SET reactions. Nevertheless, ZnO’s surface chemistry, particularly the presence of oxygen vacancies and catalytic sites, enables moderate interaction with free radicals, offering redox buffering capacity alongside well-established antimicrobial and anti-inflammatory effects. Various studies have evaluated the antioxidant activity of green-synthesized ZnO nanoparticles, where the reported IC_50_ values are attributed to the incorporation of plant secondary metabolites, specifically the phenolic compounds of the extracts. The green synthesis of ZnO nanoparticles using *C. abyssinica* tuber extract, Olive fruit extract, and *Ailanthus altissima* leaf extract has shown IC_50_ values of 127.74 [72], 87.04 [73], and 78.23 µg/ mL [74], respectively. Albarakaty et al. [75] have reported that the IC_50_ value (62 µg/mL) of ZnO nanooparticles synthesized using seed extract of *Moringa oleifera* was higher than that of the pure seed extract’s IC_50_, suggesting the contribution of the ZnO metallic structure to the antioxidant activity. Thus, the results obtained in the current study for the ZnO nanoparticles are lower than those of the reported values in the literature due to the differences in the synthesis method. Mechanistically, the purple-to-yellow color change in the DPPH assay confirms the successful reduction of the DPPH radical (Z^•^) to its non-radical form (ZH) by antioxidant agents (AH), following the stoichiometries exemplified by reactions with thiols (e.g., RSH) and polyphenols. Ascorbic acid, which is a potent antioxidant agent, was used as the positive control. As reported in S3 Fig DPPH radical scavenging ability of ascorbic acid was significantly higher than that of the tested samples, with an IC_50_ value of 4.21 µg/mL. In the context of wound healing, these antioxidant properties are critical, as oxidative stress impairs collagen synthesis, delays fibroblast migration, and perpetuates chronic inflammation. By mitigating oxidative injury, the tested components foster an optimal wound microenvironment: *Aloe vera* by directly quenching radicals and modulating cytokine release, papaya latex by enzymatically removing oxidized biomolecules and stimulating tissue regeneration, and ZnO by supporting redox balance while concurrently inhibiting microbial growth. However, the petroleum jelly in the ointment base is insoluble in the solvent system used in the assay. This physical entrapment of the active ingredients has prevented the antioxidant agents’ interactions with the DPPH free radicals. Even though the results of the ointment base’s RSA% have not demonstrated a positive activity, the above findings suggest that integrating *Aloe Vera* gel, *Carica papaya* latex, and ZnO into a single formulation could harness their individual RSA to create a synergistic therapeutic capable of reducing oxidative stress, enhancing cellular repair processes, and preventing secondary infections. Future in vivo studies should aim to elucidate their combined efficacy and optimize their ratios for maximal redox modulation and tissue regeneration [76]. Observed IC₅₀ values for *Aloe vera* gel (312.5 µg/mL) and papaya latex (454.5 µg/mL) are consistent with recent reports showing solvent system and extraction method significantly influence antioxidant efficacy [22]. The lower activity of ZnO nanoparticles (490.8 µg/mL) compared to green-synthesized ZnO in other studies reflects differences in synthesis routes, as phytochemical-mediated synthesis typically enhances radical scavenging through surface functionalization with phenolic compounds [77].

### 3.6. Whole blood assays

The whole blood assays conducted in this study offer a comprehensive evaluation of the hemocompatibility and hemostatic properties of a wound-healing ointment base and its components, ZnO nanoparticles, cassava starch, and petroleum jelly. These assays, Whole Blood Coagulation (Blood Clotting Index), Erythrocyte Adsorption, Platelet Adhesion, Hemolysis, and Clotting Time, collectively elucidate the interactions between the materials and key blood components, providing mechanistic insights into their biomedical suitability.

#### 3.6.1. Whole blood coagulation assay (blood clotting index, %BCI).

Blood clotting index quantifies the clot formation ability of the biomaterials. Higher absorbance in the supernatant, which is related with the higher BCI%, indicates poor clotting ability. The whole blood coagulation assay results (summarized in Table 1) demonstrate a significant enhancement in hemostatic potential by the ointment base comprising ZnO nanoparticles, cassava starch, and petroleum jelly, with a blood clotting index (BCI) of 3.57 ± 6.09%. This is markedly lower than the BCI values observed for the individual components-ZnO (22.26 ± 1.09%), cassava starch (8.09 ± 3.40%), and petroleum jelly (7.92 ± 1.55%)-indicating a pronounced synergistic effect when combined. BCI values under 50% indicate the suitability of the test compound to act as a better hemostatic agent with pro-coagulant effects [78]. ZnO nanoparticles are documented for their antimicrobial and pro-coagulant properties, primarily due to their capacity to induce platelet aggregation and activation of the intrinsic coagulation pathway; however, the high standard deviation suggests variability in clotting performance, potentially due to nanoparticle aggregation or dispersion issues. According to Permyakova et al [79] increasing ZnO content has contributed to increase in BCI% of the curdlan/chitosan dressings from 14 ± 5% (ZnO free) to 32 ± 5%(CUR/CS_ZnO-7%), suggesting increasing ZnO can weaken hemostatic performance. Cassava starch, a natural polysaccharide, likely contributes to hemostasis via physical absorption of plasma, concentrating clotting factors at the site, and by providing a scaffold that supports platelet adhesion and aggregation. Petroleum jelly, although inert and hydrophobic, may enhance clot retention and barrier formation by stabilizing the formulation’s adherence to the wound site. Although there are no reported BCI data for the cassava starch and petroleum jelly, starch-based nano-microporous particles were able to reduce the weight of blood loss and bleeding time [80], whereas Yim et al have reported Vaseline gauze packing as a successful measure for bleeding control in acute hemorrhagic rectal ulcer [81], by acting as barrier and moisture‑retaining agent rather than enhancing clot formation. *Carica papaya* latex and *Aloe Vera* gel have been reported for their anti-coagulant activity. Activity of papain has been characterized using fibrino(geno)lytic, anticoagulant, and antithrombotic activities [82]. Results have demonstrated that papain induces dose-dependent blood clot lysis, cleaves fibrinogen chains, and significantly prolongs prothrombin time and activated partial thromboplastin time, highlighting its’ anti-coagulant and antithrombotic activity. The BCI values were not recorded for the full formulation with active ingredients due to their anti-coagulant effects. Thus, future studies should focus on using in-vivo bleeding models to quantify the activity of the ointment to determine the optimum concentration of active ingredients to promote coagulation.

**Table 1:** Blood assay results summary.

	Whole Blood Coagulation	Erythrocyte Adsorption	Platelet Adhesion	Hemolysis Assay			Clotting Blood Time
				10mg/mL	20mg/mL	40 mg/mL	
Ointment base	3.57 ± 6.09	84.1 ± 1.09	61.55 ± 1.09	2.31 ± 1.09	4.72 ± 1.09	3.51 ± 1.09	13.30 s
ZnO NPs	22.26 ± 1.09	8.34 ± 5.10	29.1 ± 4.08	6.67 ± 3.09	5.68 ± 4.03	8.83 ± 2.09	18.34 min
Cassava starch	8.09 ± 3.40	41.1 ± 3.71	53.97 ± 2.40	6.56 ± 2.09	5.4 ± 3.15	5.14 ± 0.40	48.34 s
Petroleum jelly	7.92 ± 1.55	21.6 ± 0.55	48.22 ± 1.67	8.5 ± 1.20	7.9 ± 1.02	6.78 ± 0.54	13.34s

Recent studies confirm the hemostatic efficacy of ZnO-containing formulations. A 2025 ZnO/chitosan/tannic acid sponge significantly reduced blood clotting time from 349 s to 49 s, outperforming Celox™ (182 s) [83]. Similarly, ZnO-functionalized nanofibrous microspheres exhibited a blood clotting index of 22.82% [84]. These findings align with our observation of a low BCI (3.57%) for the composite ointment base, validating the synergistic pro-coagulant effect of ZnO with polysaccharide and hydrophobic components.

Nevertheless, significantly lower BCI of the composite ointment base suggests that the interplay among these components facilitates rapid clot formation by combining the clot-promoting effects of ZnO, higher absorbent and platelet/ coagulation-factor aggregation effects of cassava starch, with the occlusive and moisture-retaining properties of petroleum jelly, which together create an optimal microenvironment for coagulation. Furthermore, the formulation’s structure may ensure better localization and sustained contact of active components with blood, accelerating clot initiation and stabilization. These findings underscore the formulation’s potential as an effective topical hemostatic agent, especially in settings requiring rapid control of bleeding.

#### 3.6.2. Erythrocyte adsorption (RBC attachment).

The remaining RBC % as a fraction of total input RBC is expressed in the assay. Potent hemostatic dressings are often related to higher RBC attachment, as RBCs help in the formation of a stable clot at the dressing surface [85]. The erythrocyte adsorption assay results (shown in Table 1) reveal a markedly enhanced red blood cell (RBC) attachment for the ointment base (84.1 ± 1.09%) in comparison to its individual components-ZnO nanoparticles (8.34 ± 5.10%), cassava starch (41.1 ± 3.71%), and petroleum jelly (21.6 ± 0.55%)-highlighting a robust synergistic interaction that facilitates cellular adherence. Erythrocyte adsorption is critical in the early stages of hemostasis as it supports the development of a stable clot and contributes to sealing the wound site. Cassava starch plays a notable role due to its porous, hydrophilic nature that favors cellular entrapment and fluid absorption, thereby aiding in concentrating blood components at the injury interface. The relatively moderate RBC adherence seen with starch (41.1%) supports its role as a scaffold for cell immobilization. Self-gelling starch microparticles (aldehyde‐ and catechol‐modified starch) demonstrated a rapid hemostasis by accumulation of blood cells, absorbing plasma, and forming a tissue‐adhesive hydrogel in situ [86]. ZnO nanoparticles, while renowned for pro-coagulant and antimicrobial properties, showed minimal RBC attachment, likely due to their poor hemocompatibility or surface characteristics that do not favor erythrocyte interaction; the large standard deviation further suggests inconsistent cell-nanoparticle interactions. ZnO nanoparticles exhibited a size and media-dependent hemolytic effect on RBC rather than stable adhesion [87]. Therefore, RBCs exposed to ZnO sites are more likely to be damaged and washed away during the assay rather than remain adhered to the surface. Petroleum jelly, being hydrophobic and inert, likely impedes direct RBC contact with the surface, resulting in lower attachment. The low-moderate value is due to the occlusive and protective role of the compound, as it is believed to control bleeding via mechanical tamponade rather than actively participating in RBC attachment. The ability to retain RBCs and facilitate clot formation was only reported for the ointment base and its components, considering the anti-coagulant properties of the active ingredients as described. The ointment base’s superior performance may be attributed to the combined physical and chemical properties imparted by its constituents: starch enhances absorption and surface area for cell capture, ZnO may indirectly contribute by promoting protein adsorption that facilitates cell binding, and petroleum jelly aids in forming a cohesive matrix that traps cells more effectively. Thus, the formulation’s composite structure provides an optimized interface for erythrocyte adhesion, crucial for initiating clot formation and creating an occlusive layer at the wound site, making it a highly favorable candidate for wound healing applications.

Recent studies confirm the RBC attachment efficacy of ZnO-polysaccharide composites. Ma et al. reported that porous SiO₂/ZnO-carboxymethyl cellulose composite hydrogels facilitated RBC adhesion and activation, achieving a clotting time of 98 ± 18 s [88]. Liu et al. demonstrated that starch-based multilayer microparticles possess unique properties of platelet adhesion and RBC aggregation, resulting in optimal hemostatic performance [89]. These findings align with our observation of enhanced RBC attachment (84.1%) for the ointment base.

#### 3.6.3. Platelet adhesion.

Platelets are key components in stable clot formation, and the higher platelet adhesion is considered desirable for hemostatic dressings. Higher platelet adhesion % indicates higher retention of platelets on the material, which is associated with better hemostatic performance [90]. As reported by Chinnappan et al., papaya leaf extract can directly act on the reduction of platelet aggregation [91], and *Aloe* was associated with altered clotting, due to the reported intraoperative bleeding after consumption of *Aloe* tablets [92]. Hence, the assay was conducted only for the components in the ointment base to evaluate their hemostatic properties.

The platelet adhesion assay reveals a notably elevated percentage of platelet attachment (Table 1) in the ointment base (61.55 ± 1.09%) compared to its individual components-ZnO (29.1 ± 4.08%), cassava starch (53.97 ± 2.40%), and petroleum jelly (48.22 ± 1.67%), indicating a synergistic interaction that significantly augments the ointment’s pro-hemostatic potential. Platelet adhesion is a pivotal step in primary hemostasis, serving as the foundation for clot formation via subsequent platelet activation and aggregation. ZnO nanoparticles are reported to exhibit a rapid blood clotting time with better hemostatic activity when embedded into the alginate hydrogel composite bandage, compared to the alginate alone dressings [93]. In the present study, ZnO nanoparticles exhibit moderate platelet adherence (29.1%), which aligns with their known capacity to activate platelets through oxidative stress-mediated signaling and surface interactions; however, their relatively low adhesion may be due to limited surface area or aggregation, reducing effective contact. The surface morphology of the material, including roughness, porous and hydrophobic nature, also affects the platelet adhesion property [85]. Conversely, information regarding the effect of ZnO nanoparticles on platelet function is too rare to draw a conclusion about the activity. Cassava starch demonstrates substantial platelet adherence (53.97%), likely owing to its hydrophilic, porous nature that supports surface interactions and mimics the extracellular matrix, facilitating platelet capture. Starch-based shape memory sponges facilitated the hemostasis and demonstrated lower BCI via activating platelets and promoting the release of coagulation factors [94]. ZnO-loaded chitosan-sericin nano-biocomposite has also elucidated enhanced platelet interactions and better BCI [95]. Petroleum jelly, although non-bioactive, shows considerable platelet adhesion (48.22%), which may result from its ability to immobilize platelets through mechanical entrapment within its semi-solid matrix. Furthermore, petroleum products are a type of non-adherent wound care material that is designed to protect the wound site, minimizing the adherence to tissues. Interestingly, the significantly higher adhesion in the ointment base suggests an optimized surface architecture and chemical microenvironment that supports platelet localization. The interaction between starch’s absorptive scaffold, ZnO’s pro-coagulant potential, and the occlusive property of petroleum jelly likely contributes to creating a conducive surface that mimics the physiological conditions necessary for hemostasis. Moreover, the consistent and low standard deviation in the ointment base underscores the formulation’s reproducibility and stability in promoting platelet interaction. This enhanced platelet adhesion, in conjunction with high erythrocyte attachment and clotting index, underscores the ointment base’s effectiveness in rapidly initiating and sustaining hemostatic responses, thus making it a promising candidate for advanced wound care and bleeding control applications.

There are studies that confirm that ZnO-polysaccharide composites enhance platelet adhesion. A ZnO/chitosan/tannic acid sponge demonstrated superior platelet adhesion and reduced clotting time [83]. Liu et al. reported that starch-based microparticles possess unique platelet adhesion and activation properties [89]. These findings align with our observed platelet adhesion of 61.55% for the composite ointment base.

#### 3.6.4. Hemolysis assay.

Hemolysis assay was conducted for the components of the carrier system to eliminate the RBC toxicity that can be associated with the ZnO nanoparticles, cassava starch, and petroleum jelly. More RBC membrane damage results in higher absorbance (higher hemolysis %) indicate poorer hemocompatibility. Hemolysis % is accepted to be under 5% (5–10%) for a material to be considered as a hemostatic biomaterial [96]. *Carica papaya* latex and *Aloe Vera* were claimed to reduce the platelet/RBC adhesion and increase BCI, but they have also reported to stabilize erythrocyte membranes and thereby indicate no to low hemolytic profiles. Papaya leaf extract shows erythrocyte stabilization activity, prevents hemolysis, and directly acts upon platelets, specifically in conditions like dengue fever [97,98]. Herculano et al have reported that *Aloe vera*-loaded natural rubber dressings are non-hemolytic, supporting the hemocompatibility of the compound [99]. Thus, it is evident that anticoagulant activity does not necessarily indicate that the samples are attributed with RBC toxicity. The active ingredients were eliminated from the assay but not considered to cause RBC toxicity in the prepared ointment.

The hemolysis assay results across different concentrations (10, 20, and 40 mg/mL) (summarized in Table 1) demonstrate that the ointment base exhibits consistently low hemolytic activity, with values well below the generally accepted cytotoxic threshold of 5% hemolysis, particularly at 10 mg/mL (2.31 ± 1.09%) and 40 mg/mL (3.51 ± 1.09%). At 20 mg/mL, the hemolysis marginally approaches the threshold (4.72 ± 1.09%) but remains within safe limits, indicating excellent hemocompatibility. Comparatively, individual components, especially ZnO and petroleum jelly, exhibit higher and more variable hemolysis rates across concentrations. ZnO demonstrates moderate to high hemolysis (6.67% at 10 mg/mL and peaking at 8.83% at 40 mg/mL), consistent with reports that ZnO nanoparticles can disrupt erythrocyte membranes through oxidative stress or direct membrane interaction. As reported by Babu et al., ZnO nanoparticles show hemolytic activity based on the size, concnetration and time of exposure. Results of the study indicate that ZnO nanoparticles <50 nm in size dispersed in water have higher hemolytic activity compared to other sizes, and the effect was increased with increasing concentration in a time-dependent manner [87]. Even though the individual components showed and dose dependent increment in the hemolytic activity, incorporation into the ointment base has lowered the activity to the acceptable range. Cassava starch shows a decrease in hemolysis activity with concentration ranging between 6.56%(10 mg/mL) and 5.14% (40 mg/mL), suggesting a mild membrane-disruptive potential, possibly due to surface charge effects or osmotic imbalances created by the polysaccharide matrix. A boronic acid-modified thiol starch sponge had shown low hemolysis, confirming the hemostatic activity and hemocompatibility [100]. Starch is considered a highly biocompatible agent, and it is widely used in hemostats [80]. Petroleum jelly, though often considered inert, reveals a dose-dependent reduction in hemolytic values from 8.5% (10 mg/mL) to 6.78% (40 mg/mL), potentially due to physical disruption or hydrophobic interactions with erythrocyte membranes. Petroleum jelly-based dressings are used for their non-adhesive and moisture retention properties. It is clinically used and accepted widely, supporting the fact with a lack of data reported for RBC toxicity. The formulation’s lower hemolytic profile compared to its constituents indicates a significant reduction in cytotoxicity due to synergistic buffering interactions; for instance, the hydrophilic starch matrix may shield erythrocytes from direct ZnO or petroleum jelly contact, while the ointment’s semi-solid structure might reduce nanoparticle aggregation and mobility. Notably, the stable hemolysis profile across all tested concentrations underscores the ointment base’s safety and reliability for topical application, reinforcing its suitability for hemostatic use without causing undue erythrocyte damage or hemolytic toxicity.

Recent studies confirm the hemocompatibility of ZnO-starch systems. Cabo-Araoz et al. reported that cassava starch-based scaffolds exhibited hemolysis levels below 2% [101]. Similarly, a study on ZnO-loaded chi

tosan nanoparticles demonstrated hemolysis below 5%, establishing their hemocompatibility for biomedical use [102]. These findings align with our observation of low hemolysis (2.31–4.72%) for the ointment base.

#### 3.6.5. Clotting blood time.

Clotting blood time was used further evaluate the hemostatic performance of the carrier system. In the assay shorter clotting time relative to the blank indicates improved coagulation. The pro-hemostatic properties of the carrier were characterized in the current assay [103]. Enzymes present in the papaya latex are responsible for the prolonged clotting time and have shown anticoagulant activity in animal models [104]. Furthermore, Cordiers & Steenkamp listed papaya as a plant material that alters coagulation [92]. Additionally, cold aqueous *Aloe vera* extract has exhibited an extended mean clotting time of 18.67 ± 1.47 min, surpassing the normal 5–15 min range, which confirms the delayed coagulation effects in-vitro [105]. Therefore, active ingredients with proven extended coagulation times were not considered for the assay.

The clotting time assay results (Table 1) reveal a dramatic reduction in coagulation time for the ointment base (13.30 seconds) relative to the negative control (300 seconds), indicating a potent pro-coagulant effect. Similar drastic reduction in clotting time has been reported for the tannic acid-coated gauze immobilized with thrombin, where the clotting time reduced from 563 ± 32 s (control) to 87 ± 12 s [106]. Among the individual components, petroleum jelly (13.34 seconds) and cassava starch (48.34 seconds) also exhibit clot-promoting properties, whereas ZnO nanoparticles significantly prolong clotting time (18.34 minutes), suggesting an inhibitory or non-contributory role in isolation. The rapid clotting seen with the ointment base, nearly equivalent to petroleum jelly alone, suggests that the formulation’s matrix effectively promotes hemostasis through both physical and biochemical mechanisms. Petroleum jelly likely contributed by forming an occlusive barrier that minimizes blood flow and preserves clotting factor concentration at the wound site, facilitating rapid clot initiation. Biologically inert petroleum jelly provided a pro-coagulant environment in this experimental setting. Cassava starch, with its hydrophilic and absorptive properties, concentrates clotting proteins and cellular components, enhancing the clotting cascade. Boronic acid-modified thiol starch sponge has reached complete hemostasis in ~61.5 s on rabbit liver in a study conducted by W. Huang et al [100]. In contrast, the unexpectedly prolonged clotting time with ZnO may stem from nanoparticle interference with enzymatic elements of the coagulation cascade, possible oxidative stress effects, or poor dispersion that limits effective surface interaction. J. Y. Yang et al. demonstrated that ZnO nanoparticles are responsible for delayed clotting time and thrombin generation. According to the results, ZnO in the 20 and 100 nm sizes, with various surface chemistries has contributed to prolonged partial thromboplastin time (aPTT) and prothrombin time (PT) in dose dependent manner [107]. Conversely, ZnO has reported to have both anti-coagulant and pro-hemostatic activities. The prolonged aPTT and PT times were considerably longer in platelet-poor plasma, as ZnO inhibits the activity of some coagulation factors. ZnO’s hemostatic activity is dose-dependent, as shown by Permyakova et al [79]. When ZnO was incorporated in low concentrations into materials like PEG hydrogels, it reduced the clotting time from 193 s (PEG alone) to ~60 s [108]. While ZnO as an individual component exhibits a higher coagulation time in the study, it has contributed to the lower coagulation time of the ointment in the composite. Hence, it can be safely concluded that, when incorporated into the composite ointment, ZnO’s negative impact appears neutralized or modulated by the synergistic interactions with starch and petroleum jelly. The overall efficacy of the ointment base likely arises from a well-balanced structural and chemical environment that accelerates platelet aggregation, RBC and protein adsorption, and fibrin mesh formation, effectively reducing clotting time by over 95% compared to the control. These findings strongly support the formulation’s potential application in clinical and emergency wound care settings where rapid hemostasis and coagulation are crucial.

Previous studies confirm the hemostatic efficacy of ZnO-starch composite systems. A ZnO/chitosan/tannic acid sponge achieved a clotting time of 49 s compared to 349 s for the control [83], while ZnO-loaded chitosan nanoparticles demonstrated significantly enhanced hemostatic activity [102]. These findings align with our observed clotting time reduction from 300 s (control) to 13.3 s for the ointment base.

Together, these assays highlight the differential yet complementary roles of each component. ZnO nanoparticles offer strong pro-coagulant and bio-interactive potential but must be carefully dosed due to hemolytic risk at higher concentrations. Cassava starch provides a biodegradable scaffold that supports erythrocyte and platelet interactions without compromising biocompatibility. Petroleum jelly and the ointment base serve as a stable platform for clot formation and a physical barrier in moisture retention, creating an optimal environment. The ointment base emerges as a biocompatible, hemostatically active vehicle that can significantly enhance hemostatic properties when synergistically formulated with ZnO nanoparticles and cassava starch. These results support the integration of these materials into multifunctional ointments designed for optimal bleeding control, infection management, and tissue regeneration.

### 3.7. Protease assay

- Tyrosine equivalents released = 318.56 µM x 11 (total volume of sample)
- Enzyme activity = 3504.16/ (10 x 1 x 3)
- Enzyme activity = 116.50 (Unit/ mL enzyme)
- Solid sample = 116.50 units mL^-1^/ 2 mg mL^-1^
- **Protease activity of papain sample = 58.40 units/mg of the solid**

The findings of this study establish the potent proteolytic capacity of *Carica papaya* latex powder, validating its functional suitability for biomedical applications, particularly in enzymatic debriding [109]. Absorbance values of *Carica papaya* latex powder in the protease assay are shown in Table 2. Utilizing a non-specific protease assay with casein as a substrate, the assay demonstrated a clear concentration-dependent increase in enzymatic activity, as evidenced by the progressive release of tyrosine (S4 Fig) quantified via the Folin-Ciocalteu method at 660 nm. A tyrosine standard curve is commonly used in protease activity assays to quantify the amount of tyrosine released from protein substrates during enzymatic hydrolysis. By plotting known concentrations of tyrosine against their corresponding absorbance values, a linear calibration curve is generated, which enables the estimation of protease activity based on the absorbance of unknown samples. The linearity of the standard curve reinforced the reliability of the assay, while the derived activity values confirmed a direct correlation between enzyme concentration and proteolytic efficiency. Mechanistically, the observed activity is attributed to cysteine proteases, predominantly papain, which mediate peptide bond hydrolysis through a catalytic triad of cysteine, histidine, and asparagine residues [110]. This catalytic mechanism facilitates the breakdown of proteinaceous substrates such as casein, mimicking the enzymatic degradation of necrotic tissue encountered in chronic wounds. Importantly, this proteolytic function serves a dual purpose in wound healing: it promotes enzymatic debridement by clearing denatured proteins and fibrin slough, while also supporting extracellular matrix remodeling, thereby enhancing granulation tissue formation and epithelialization. Glahn et al. has reported the removal of thermally damaged coagulation zone tissues following ablative fractional laser treatment using papain-urea, which highlights papain’s activity in targeted proteolysis of necrotic tissue [111]. The effect of *Carica papaya* extract in incised wounds was reported with the decrease in the wound length starting from the 4^th^ day of the application, consistent with the tested concentration range (25%−75%). Further histological observations confirmed the enzymatic debridement and collagen turnover effects, which improved the epithelialization [112]. The concentration-dependent kinetics suggest that even low doses of the latex powder may be therapeutically effective, a valuable trait for minimizing potential cytotoxicity in topical formulations. Additionally, the natural origin and biocompatibility of *Carica papaya* latex enhance its translational potential in clinical settings, especially as an alternative to synthetic or animal-derived proteases. These results are in agreement with prior studies highlighting the strong proteolytic and wound-modulating effects of papain-based treatments [29]. Nevertheless, for clinical integration, further research is warranted to address scalability, stability, and formulation optimization. In vivo studies are essential to validate the bioactivity and safety of the latex under physiological conditions, and its integration into hydrogel-based or polymeric delivery systems may further augment therapeutic efficacy. Overall, the study provides compelling evidence for the use of *Carica papaya* latex as a multifunctional biotherapeutic agent in enzymatic debridement and tissue regeneration [113].

**Table 2:** Absorbance values of *Carica papaya* latex powder in the protease assay.

Concentration (μg/ml)	Average Absorbance	SD	SE
1000	0.61	0.04	0.02
500	0.57	0.02	0.02
250	0.49	0.04	0.02
125	0.45	0.04	0.02
62.5	0.42	0.07	0.04
31.25	0.35	0.08	0.05
15.625	0.32	0.08	0.04

A protease activity of 58.40 units/mg for papaya latex is considered moderate to high, depending on the source and purity of the enzyme. Commercial preparations typically show a wide activity range, from 10 to over 100 units/mg, depending on factors like assay conditions (e.g., pH, temperature), substrate used, and the degree of purification. The above result suggests that the sample has a substantial enzymatic capacity, indicating good functionality for general proteolytic applications. However, for pharmaceutical or high-precision industrial use, higher specific activities or further purification might be required to meet stringent standards. Recent studies confirm the proteolytic efficacy of *Carica papaya* latex. Glahn et al. demonstrated that papain–urea treatment achieved a 44% reduction in the coagulation zone of ex vivo skin samples, highlighting papain’s targeted proteolysis of denatured tissue [114]. Badgujar and Mahajan reported that *Carica papaya* latex exhibits high proteolytic activity, with cysteine proteases (predominantly papain) as the active enzymes [115]. The measured activity of 58.40 units/mg for our papaya latex powder falls within the range of commercial papain preparations (≥8–30 U/mg), confirming its substantial enzymatic capacity.

### 3.8. Antibacterial activity

#### 3.8.1. Agar well diffusion assay.

**Fig 5:**
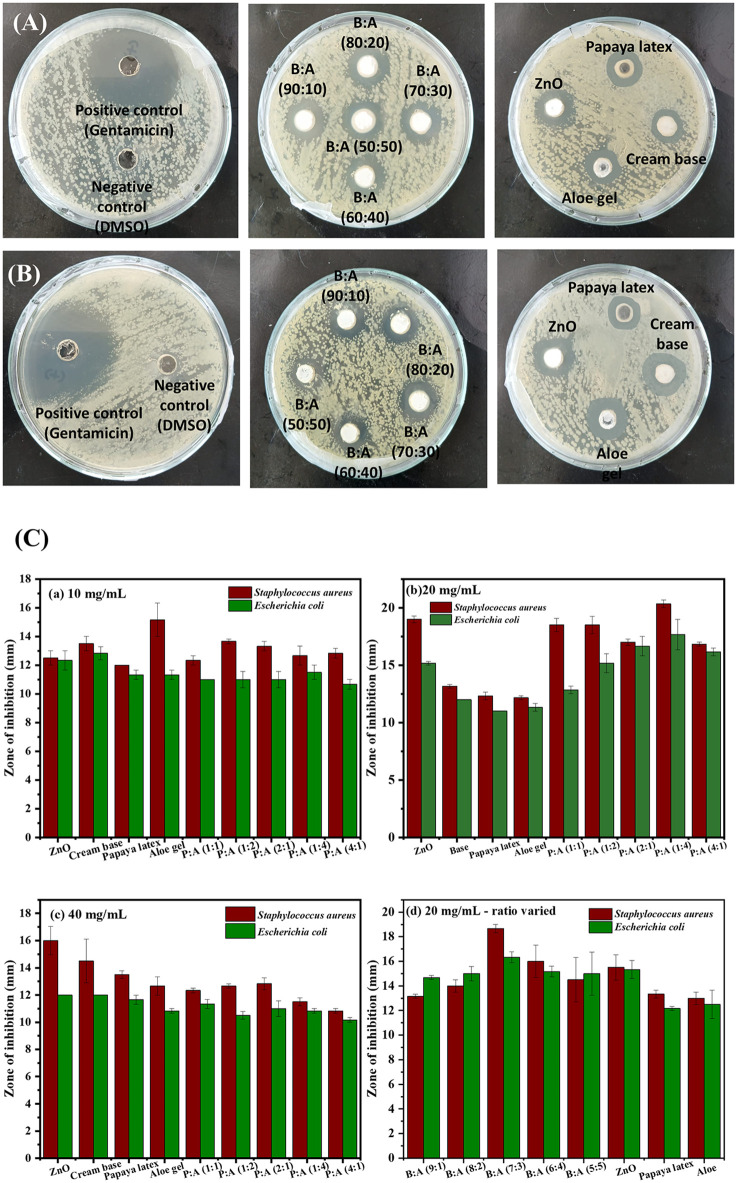
Zones of Inhibition against (A) *Staphylococcus aureus* (B) *Escherichia coli* (C)Variation of the zones of inhibition (in mm) against the test organisms by the tested samples at (a) 10 mg/mL, (b) 20 mg/mL, (c) 40 mg/mL (d) varied cream base ratios at 20 mg/mL.

The tropical ointment synthesized in the current study consists of ZnO nanoparticles, petroleum jelly, and cassava starch as the base ingredients and *Carica papaya* latex and *Aloe vera* gel as active ingredients. The antibacterial activity of the above components was tested against the Gram-positive *Staphylococcus aureus* and Gram-negative *Escherichia coli* bacterial strains under varied concentrations (Fig 5 (A) and (B)). ZnO nanoparticles are one of the most widely used metal oxide nanoparticles due to their superior antibacterial properties. They are known to induce oxidative stress by generating reactive oxygen species (ROS), leading to lipid peroxidation, protein denaturation, and DNA damage. Additionally, ZnO nanoparticles can penetrate bacterial cell membranes, causing leakage of intracellular components and loss of membrane integrity [116]. Petroleum jelly is described as an occlusive, inert barrier which prevent the bacterial interactions with the wound site rather than providing antimicrobial activity via biological mechanisms [117]. Studies on starch-based material used in wound-care suggest that the antibacterial activity of the materials is attributed to the activity of the incorporated compounds, while starch alone had no significant activity against bacterial pathogens [117,118]. Cassava starch and petroleum jelly were added as the components of the ointment base, grounded on their biocompatibility and added advantages in wound care as described in section 3.5. Thus, the antibacterial activity of the cassava starch and petroleum jelly was not evaluated in the current study.

*Aloe barbadensis Miller* gel and the papaya latex have gained interest in the wound care studies mainly due to their antibacterial activity and proteolytic activity, respectively. Even though available literature does not provide satisfactory data for the antibacterial activity of the *Carica papaya* latex, various studies have highlighted the use of *Carica papaya* latex as a bio-reductant in nanoparticle synthesis. In the study conducted by Chandrasekaran et al., silver nanoparticles have been synthesized using *Carica papaya* latex, and they have shown enhanced antibacterial activity againts Gram positive *E. faecalis*, *B. subtilis* [119]. The antibacterial activity of *Aloe vera* is attributed to the presence of bioactive compounds, including anthraquinones such as aloe-emodin, saponins, and phenols. They were reported to function by disrupting the bacterial cell walls, increasing membrane permeability, inhibiting the enzymes in metabolic pathways, and interfering with the biofilm formation of the pathogenic bacteria [120]. Therefore, the novel formula is expected demonstrate an enhanced antibacterial activity with the incorporation of the proven, potent antibacterial agents, including ZnO and *Aloe barbadensis Miller* gel.

The antimicrobial evaluation of the novel ointment incorporating ZnO nanoparticles, cassava starch, white petroleum jelly, *Carica papaya* latex, and *Aloe barbadensis miller* gel demonstrated promising activity against Escherichia coli and Staphylococcus aureus, as assessed by the agar well-diffusion assay (Fig 5(A) and 5 (B)). The experimental design varied the ratios of active ingredients and base to determine their individual and synergistic effects. The variation of the zones of inhibition (in mm) against the test organisms by the tested samples at (a) 10 mg/mL, (b) 20 mg/mL, (c) 40 mg/mL, and (d) varied ointment base ratios at 20 mg/mL is shown in Fig 5 (C).

A clear dose-dependent increase in antibacterial activity was observed for ZnO against *Escherichia coli*, where inhibition zones increased progressively with concentration as 12.5 ± 0.50 mm (10 mg/mL), 15.17 ± 0.17 mm (20 mg/mL), and 16 ± 1.04 mm (40 mg/mL). A similar observation was reported by Jalal et al, where antibacterial activity against *Escherichia coli* increased with concentration. The study has confirmed the production of higher H_2_O_2_ on the surface, making it lethal to bacteria. [121]. Similar inhibition zones were reported. Conversely, for *S. aureus*, the highest inhibition was noted at 20 mg/mL (19 ± 0.29 mm), with 10 mg/mL (12.33 ± 0.67 mm) and 40 mg/mL (12 ± 0.00 mm) showing comparable activity, suggesting an optimum antibacterial concentration near 20 mg/mL. Gram-positive bacteria are more susceptible to the antibacterial activity of ZnO due to the absence of an outer membrane, like *Escherichia coli*. However, once the critical surface dose is reached, an increase in the ZnO concentration results in nanoparticle aggregation, reducing the diffusion. Therefore, no enhancement in the antibacterial activity was reported for the ZnO at 40 mg/mL. The saturation effect of the *Staphylococcus aureus* is consistent with the reports of Lallo da Silva et al [122]. ZnO nanoparticles have indicated significant antibacterial activity against the tested bacterial strains. ROS such as hydroxyl radicals, superoxide, and hydrogen peroxide are generated via the photocatalytic reaction of ZnO nanoparticles. These free radicals can remain at the bacterial surface or can diffuse through the cell wall, thereby causing oxidation in the lipids, proteins, and DNA. The activity of the free radicals contributes to the membrane damage, leakage of intracellular contents, and ultimately to cell death. Additionally, partial dissolution of ZnO releases Zn^2+^, which can interfere with bacterial amino acid metabolism, enzyme systems, and active transport mechanisms [123]. The positive surface charge of the ZnO nanoparticles facilitates their binding with the generally negatively charged cell membrane of the bacterial cells. These interactions cause mechanical disruption of the cell membranes as reported by the SEM and TEM image analysis [124]. The collective action of these mechanisms contributes to the antibacterial activity of the ZnO nanoparticles.

*Carica papaya* latex has exhibited moderate antibacterial activity against *Escherichia coli*, with no consistent dose-dependent trend, 12 ± 0.00 mm (10 mg/mL), 11 ± 0.00 mm (20 mg/mL), and 13.5 ± 0.29 mm (40 mg/mL). The average diameter of the inhibition zones varied from 11.33 ± 0.33 mm at 10 mg/mL to 12.33 ± 0.33 mm at 20 mg/mL for *S. aureus*. Overall, *Carica papaya* latex has shown moderate antibacterial activity at the tested concentration range. Chinasa et al have reported the antibacterial activity of *Carica papaya* latex against *Bacillus subtilis*, *E coli,* and *S. aureus*. The reported inhibition zones were in the range of 6–9 mm (0.25–0.5 mg/mL) and1 3–17 mm (1–2.5 mg/mL) for *Escherichia coli* and 4–7 mm (0.25–1.5 mg/mL) and 9.1–13.1 mm (2–2.5 mg/mL) for *Staphylococcus aureus* [125]. According to their claims antibacterial activity of the papaya latex is attributed to the higher content of flavonoids, tannins, alkaloids, and terpenes. Proteolytic enzymes including papain, chymopapain A & B, papaya peptidase A, and carpaine are believed to interfere with the microorganism growth. Flavonoids act on the cell wall and extracellular proteins, and tannins can obstruct the microbial adhesion enzymes, causing detrimental effects on the growth cycle of the bacteria.

*Aloe barbadensis Miller* gel, in contrast, displayed a reduction in activity with increasing concentration, showing maximum inhibition against *Escherichia coli* at 10 mg/mL (15.17 ± 1.17 mm). However, the highest inhibition for *Aloe barbadensis Miller* gel was reported at 20 mg/mL against *Staphylococcus aureus* (12.17 ± 0.17 mm). Increased viscosity of the sample is likely to contribute to reducing the diffusion of the active compounds, resulting in the lower inhibition zones at higher concentrations. Aloe vera’s antimicrobial activity is attributed to anthraquinones, saponins, and phenolic compounds, which enhance membrane permeability and interfere with bacterial metabolic pathways [126]. Aloe-emodin from *Aloe vera* extract was reported to deform the cellular morphology of *S. epidermidis* and disrupt the selective permeability of the cell wall. Transcriptional profiles of the treated cells have also revealed changes in the genes associated with biofilm formation, sulfur metabolism, L-lysine, and peptidoglycan biosynthesis [120]. A previous study has shown that *Aloe vera/*chitosan films incorporating green-synthesized ZnO nanoparticles produced inhibition zones of 13.83 mm against *Staphylococcus aureus* and 15.17 mm against *Escherichia coli* [127].

The ointment base alone exhibited moderate but considerably higher activity compared to the active ingredients. *Escherichia coli* inhibition zones varied from 13.5 ± 0.50 mm (10 mg/mL) to 14.5 ± 1.61 mm (40 mg/mL), indicating that the base formulation may possess inherent antibacterial properties of the ZnO nanoparticles at the tested concentrations. On the contrary, activity of the ointment base against *Staphylococcus aureus* showed the best activity at 20 mg/mL (13.17 ± 0.17 mm) concentration in accordance with the superior activity of the ZnO at the same concentration. Overall, *Escherichia coli* showed the greatest inhibition at higher concentrations (notably 40 mg/mL), whereas *Staphylococcus aureus* demonstrated optimal sensitivity at 20 mg/mL for most formulations.

At 10 mg/mL, combinations of *Carica papaya* latex and *Aloe barbadensis Miller* gel exhibited moderate antibacterial effects with minimal variation across ratios. The *Escherichia coli* inhibition zone ranged between 12.3 mm (1:1) and 13.7 mm (1:2), with the *Carica papaya*: *Aloe* 1:2 formulation showing slightly superior activity (13.67 ± 0.17 mm). This suggests a minor synergistic effect at balanced ratios but no significant concentration-dependent enhancement.

At 20 mg/mL, a substantial enhancement in *Escherichia coli* inhibition was observed with increasing *Aloe barbadensis Miller* gel concentration. The highest inhibition zone (17.67 ± 1.22 mm) was recorded for the *Carica papaya*: *Aloe* 1:4 ratio, while formulations with higher *Carica papaya* latex proportions (2:1 and 4:1) produced slightly lower but comparable inhibition (16.67 ± 0.83 mm and 16.17 ± 0.33 mm, respectively). This indicates that a higher *Aloe barbadensis Miller* gel content markedly improves antibacterial efficacy at this concentration. Similarly, against *S. aureus*, the 1:4 formulation exhibited the most pronounced inhibition (20.33 ± 0.33 mm), surpassing even the ZnO control (19 ± 0.29 mm). This synergy can be attributed to the antibacterial activity of *Aloe vera*, which disrupts bacterial membranes, degrades bacterial proteins, and disrupts biofilm formation, preventing bacterial adhesion and colonization [125]. Interestingly, further increasing the ointment concentration to 40 mg/mL did not proportionally enhance activity, suggesting potential saturation or inhibition effects at higher component levels.

At 40 mg/mL, the antibacterial efficacy of all *Carica papaya*: *Aloe* formulations has declined slightly compared to the 10 and 20 mg/mL. For *Escherichia coli*, the 2:1 (12.83 ± 0.44 mm) and 1:2 (12.67 ± 0.17 mm) combinations retained moderate activity, while higher *Aloe barbadensis Miller* gel concentrations have led to reduced inhibition, suggesting potential interference for the diffusion of the compound with higher concentrations. *Staphylococcus aureus* has followed a similar pattern, with the 1:1 formulation showing the highest inhibition (11.33 ± 0.33 mm).

Collectively, these findings reveal that 20 mg/mL formulations, particularly *Carica papaya*: *Aloe* 1:4, yielded the highest antibacterial potency against both *Escherichia coli* and *S. aureus*, indicating a synergistic enhancement-driven activity at intermediate doses. Hence, this ratio was selected as the optimum active ingredient ratio with the best antibacterial activity and was used to evaluate the effect of changing the base to active ingredient ratio on the antibacterial activity.

Further optimization was performed for the 20 mg/mL *Carica papaya*: *Aloe* (1:4) formulation by varying the Base: Active ingredient ratios. For *Escherichia coli*, inhibition zones increased from 14.68 ± 0.16 mm (9:1) to 16.33 ± 0.44 mm (7:3), indicating that moderate reduction of the base component improved antibacterial performance. Further reduction of the base beyond this point (6:4 and 5:5) resulted in decreased inhibition, suggesting that excessive active content may compromise diffusion or stability. Additionally, ZnO concentration was reduced in the formula with the reducing concentration of the base, suggesting the contribution of synergy between ZnO concentration and active ingredients for the enhanced antibacterial activity. A similar pattern was evident for *S. aureus*, where activity peaked at the 7:3 ratio (18.67 ± 0.33 mm) and declined at higher active contents (6:4 = 16 ± 1.32 mm; 5:5 = 14.5 ± 1.80 mm). Notably, both ZnO (19 ± 0.29 mm) and the 7:3 formulation with the 1:4 active ingredient ratio (18.67 ± 0.33 mm) demonstrated comparable inhibition, highlighting the optimized 7:3 ratio as the most effective balance between formulation integrity and antibacterial performance at 20 mg/mL.

Antibacterial activity of the novel ointment was mainly attributed to the superior antibacterial activity of ZnO nanoparticles and considerably higher antibacterial activity of the *Aloe vera* gel. However, papaya latex has shown moderate inhibition for the tested bacterial strains, though it has not significantly contributed to the enhancement of the activity in the synthesized formulations. Overall, the results underscore the importance of optimizing both active ingredient ratios and concentration to maximize antibacterial efficacy. The combination of ZnO with botanical extracts offers a promising strategy for enhancing wound care while providing broad-spectrum antimicrobial coverage. Future directions should involve mechanistic studies to elucidate the molecular basis of observed synergism and in vivo validations to establish clinical translatability.

Mohanasundaram and Saral (2025) reported that green-synthesized ZnO nanoparticles exhibited promising antimicrobial activity against pathogens, with the highest inhibition exceeding 80% in antioxidant assays [128]. Channa et al. (2025) demonstrated that phytosynthesized ZnO nanoparticles (15.94–23.32 nm) effectively inhibited *Escherichia coli* and *Staphylococcus aureus* growth, confirming their significant antimicrobial potential [129]. Additionally, a 2024 study on *Brevibacillus laterosporus* peptides revealed distinct antibacterial mechanisms against Gram-negative and Gram-positive bacteria through membrane disruption, supporting the multi-pathway activity of our ZnO–*Aloe*–papaya system [130].

#### 3.8.2. Minimum inhibitory concentration (MIC) and minimum bactericidal concentration (MBC).

To further characterize the antimicrobial efficacy of the optimized ointment formulation, comprising a base: active ingredient ratio of 70:30, with *Carica papaya* latex to *Aloe barbadensis miller* gel at 1:4, the minimum inhibitory concentration (MIC) and minimum bactericidal concentration (MBC) assays were performed against *Escherichia coli* and *Staphylococcus aureus* (Table 3). The ointment demonstrated potent bactericidal activity with MICs of 5 mg/mL and 2.5 mg/mL, respectively, and MBCs of 0.625 mg/mL for both organisms, resulting in MBC/MIC ratios of 0.125 for *Escherichia coli* and 0.25 for *Staphylococcus aureus* (Table 3). Since an MBC/MIC ratio ≤4 is indicative of bactericidal action, these values confirm the ointment’s ability not only to inhibit bacterial growth but also to eradicate the pathogens effectively [131]. Notably, the lower MIC for *Staphylococcus aureus* suggests a higher sensitivity of Gram-positive bacteria to the formulation, which may stem from differences in cell wall composition that influence permeability to the bioactive constituents [132]. The observed high potency is consistent with the synergistic interaction between ZnO nanoparticles, known for inducing oxidative stress via ROS generation and membrane disruption [133], and phytochemicals in *Aloe* vera gel, such as aloin and saponins, which interfere with microbial enzymatic systems and structural integrity [134]. Papaya latex, rich in proteolytic enzymes like papain, likely enhances penetration and degradation of bacterial proteins, further contributing to the bactericidal effect [135]. The superior performance against both Gram-negative and Gram-positive strains at relatively low concentrations suggests that the formulation could minimize reliance on conventional antibiotics, potentially reducing the risk of resistance development. Future studies should explore its in vivo efficacy, cytotoxicity, and wound healing kinetics to advance toward translational and regulatory development. ZnO NPs are highly popular for use in the pharmaceutical and biomedical industries due to the excellent response towards a wide variety of bacterial strains and are nontoxic to humans at low concentrations. According to Yabhouni et al., a chitosan/Ag/ZnO nanocomposite showed a remarkable antibacterial activity against human pathogens (*Staphylococcus aureus*, *Escherichia coli*) with the highest potency observed for *Escherichia coli* with both MIC and MBC values of 0.1562 µg/µL. A recent study has shown that papaya latex extract demonstrated a MIC of 12.5% and a MBC of 25% against *Pseudomonas aeruginosa*, confirming its potent bactericidal activity [136].

**Table 3:** MIC, MBC, and MBC/MIC ratios for the test formulation against *Escherichia coli* and Staphylococcus aureus.

	MIC (mg/mL)	MBC (mg/mL)	MBC/MIC	Effect/Activity
*Escherichia coli*	5.00	0.63	0.13	Bactericidal
*Staphylococcus aureus*	2.50	0.63	0.25	Bactericidal

#### 3.8.3. Time-kill curve analysis.

**Fig 6:**
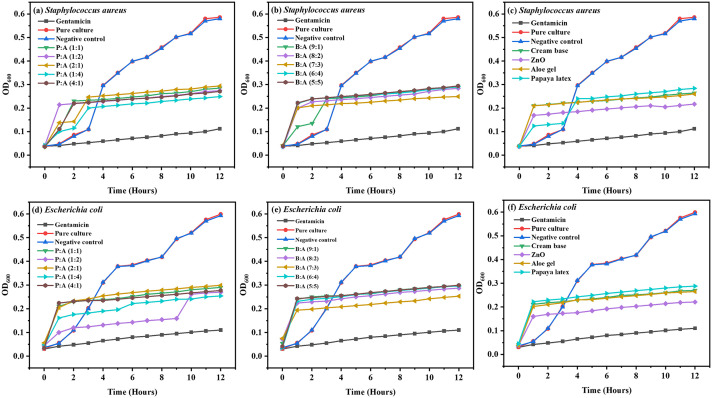
Time-kill curves of *Staphylococcus aureus* (a-c) and *Escherichia coli* (d-f) against the tested samples.

Time-kill curve assay was conducted to assess the bactericidal or bacteriostatic nature of the antimicrobial agents, in this case, the formulated ointments as well as their individual components, and their effectiveness over time. It evaluates how quickly the bacterial population declines after exposure to the antimicrobial agent. The assay can also be used to study the relationship between the concentration of an antimicrobial agent and its effect on bacterial growth [137]. The results demonstrate a gradual decline in bacteria (*Staphylococcus aureus* and *Escherichia coli*) counts over 12 hours (shown in Fig 6 (a),(b),(c) and Fig 6 (d),(e),(f), respectively), with significant reductions observed within the first six hours. The ZnO nanoparticles and ointments with a 70:30 (base: active) ratio showed the most pronounced bactericidal effects, while the ointment base alone exhibited minimal impact on bacterial viability. The control groups (gentamicin as positive control and DMSO as negative control) confirmed the assay’s validity, with gentamicin displaying the highest bacterial clearance. These results indicate that the active components work synergistically to disrupt bacterial cell viability. Bacterial survival was monitored at hourly intervals, revealing a progressive decline in colony-forming units, with the most pronounced bactericidal effect observed in the formulation containing a 1:4 ratio of *Carica papaya* latex to *Aloe barbadensis Miller* gel. Mechanistically, the observed bacterial reduction is likely driven by multiple complementary pathways: proteolytic enzymes in papaya latex (e.g., papain, chymopapain) degrade the peptidoglycan matrix, particularly targeting Gram-positive organisms like *S. aureus*; phenolic compounds and polysaccharides in *Aloe barbadensis Miller* gel compromise membrane integrity across both bacterial classes, resulting in leakage and lysis. Kaur et al. have demonstrated a significant reduction in microbial growth with *Aloe vera*-containing films after prolonged storage. ZnO nanoparticles generate reactive oxygen species (ROS), inducing oxidative damage to bacterial DNA, proteins, and lipids, especially in Gram-negative species such as *Escherichia coli*. Additionally, inhibition of protein synthesis by bioactives in the plant extracts may further attenuate bacterial viability. These mechanisms are particularly relevant to wound care, where infection control is critical to preventing chronicity and sepsis. Furthermore, its capacity to disrupt biofilms, key in chronic wound pathophysiology, highlights its therapeutic relevance. Recent time-kill studies on ZnO-containing nanocomposites confirm the bactericidal kinetics observed in our formulation. EL-Moslamy et al. (2025) demonstrated that a ternary CuO/Mn₃O₄/ZnO nanocomposite (18.5 µg/mL) achieved >97% reduction in viable counts of multidrug-resistant *Escherichia coli* and *Staphylococcus aureus* after 45 hours [130,138]. Similarly, Akinduti et al. (2021) showed that *Aloe vera* latex significantly reduced resistant *Escherichia coli* to <3.0 Log₁₀CFU/mL after 24 hours [139]. These findings align with our observed progressive decline in bacterial counts over 12 hours, with pronounced reductions within the first six hours for the ZnO and 70:30 formulations. Consistent with the literature, the results affirm the broad-spectrum antibacterial activity of the constituents. Future studies should explore efficacy against multidrug-resistant strains, validate findings through in vivo models, and refine formulation strategies to optimize therapeutic delivery and stability in clinical wound care applications.

## 4. Conclusions

The development of a topical formulation comprising *Carica papaya* latex powder, *Aloe barbadensis Miller* gel, and ZnO nanoparticles represents a novel and integrative approach to wound management by harnessing the individual and synergistic therapeutic properties of each component. This study systematically evaluated the formulation’s proteolytic, antioxidant, hemocompatible, and antibacterial properties, which are key determinants in effective wound healing. *Carica papaya* latex demonstrated robust protease activity, facilitating effective debridement through the breakdown of necrotic tissue. Concurrently, DPPH assay results revealed significant antioxidant capacity across all three constituents, with implications for mitigating oxidative stress and supporting tissue regeneration. Hemocompatibility assays confirmed the formulation’s safety, with low hemolysis rates and accelerated clotting times indicating compatibility with human blood and potential to support hemostasis. Notably, the formulation’s antibacterial efficacy was validated through a spectrum of microbiological assays, exhibiting potent activity against both *Staphylococcus aureus* and *Escherichia coli*, with low minimum inhibitory concentrations and bactericidal effects, underscoring its relevance for infection control in chronic wounds. The observed biological enhancements were particularly prominent at a 1:4 ratio of *Carica papaya* latex powder and *Aloe barbadensis Miller* gel, suggesting an optimal synergistic interplay that enhances both efficacy and biocompatibility. It is important to highlight that the study was conducted to assess the antibacterial, antioxidant, and hemostatic properties of the synthesized novel ointment and its components in vitro experimental conditions. The selected properties are key parameters to be considered in the wound healing process. As a result, the formulation’s efficacy and safety in vivo have not been confirmed. Despite this, the promising in-vitro data provide a solid basic understanding for additional research into the ointment’s therapeutic potential. Future research should emphasize refining component ratios to maximize therapeutic synergy while minimizing cytotoxicity, coupled with preclinical and clinical trials across diverse wound models. Advanced delivery platforms such as hydrogel scaffolds or polymer-based encapsulation could further augment the formulation’s stability, penetration, and sustained release.

## Supporting information

S1 Section Methodology of the whole blood assays. (DOCX)

S2 Section Raw Data. All raw data from the experiments, including blood coagulation assay, RBC attachment, platelet adhesion, hemolysis, clotting blood time, DPPH assay, protease assay, agar well diffusion assay, and time kill curve data.
(DOCX)

S1 Scheme Experimental design for the formulation, characterization, and biological evaluation of the ointment. (TIF)

S1 Fig HPLC chromatograms of extracted compounds. (TIF)

S2 Fig Bar graph for RSA of the synthesized compounds. (TIF)

S3 Fig DPPH radical scavenging activity of ascorbic acid. (TIF)

S4 Fig Tyrosine standard curve. (TIF)
