# Supplementary material for: Synergistic Bioactive Ointment: ZnO Nanoparticles Combined with Carica papaya Latex and Aloe Vera Gel for Broad-Spectrum Biomedical Applications
Source: PLoS One. 2026 Jul 21;21(7):e0353765. doi: 10.1371/journal.pone.0353765 (PMC13387519; doi:10.1371/journal.pone.0353765)
Supplement: S1 Section — (DOCX) [file pone.0353765.s001.docx]

# Methodology - Whole Blood Assays

**Whole blood coagulation assay (Blood clotting index)**

Human blood drawn from venepuncture was collected in tubes (containing sodium citrate 1:9,v/v) and stored at 5° C. A weight of 10mg of the ointment was added to a beaker along with 100 μL of anticoagulated blood and 20 μL of 0.2 M CaCl2 solution was added and placed in a water bath at 37 ^o^C. A volume of 25 mL of DI water was added after 5 minutes. Following this, the OD values were taken at 545 nm. A beaker containing all other components except for the sample was used as a control. The following equation was used to determine the blood coagulation index. The BCI values were calculated using the following formula (1):

**BCI(%) = (As/Ac)×100**

As- absorbance of the sample, Ac- absorbance of the control

**Red blood cell (RBC) attachment**

The red blood cell (RBC) attachment was evaluated as follows. First, 5% hematocrit of RBC suspension was obtained by centrifugation of citrated whole blood under 400 G for 10 min, then diluted with PBS. Next, 100 μL of diluted RBC suspension was dropped onto an ointment, followed by incubation for 1 h at 37 °C. Samples were then rinsed five times with PBS and transferred into 4 mL of DI water to lyse the attached RBCs. After 1 h at 37 °C, the OD 540 nm of RBC lysates was determined using a UV-visible spectrophotometer. The OD 540 nm value of a solution containing 100 μL of RBC suspension in 4 mL of distilled water was used as a reference value. The percentage of RBCs attached was calculated using the following formula (2):

Percentage of Red Blood Cell attachment (%RBC attachment)

**= OD gauze / OD reference x 100%**

**Platelet adhesion assay**

Platelet-rich plasma (PRP) was obtained by centrifugation of citrated whole blood under 400 G for 10 min. A total of 100 μL of PRP was dropped onto the ointment, followed by incubation for 1 h at 37 °C. Samples were then rinsed five times with PBS to remove nonadherent platelets. Thereafter, 0.6 mL of 1% Triton X-100 in PBS was added into each well to lyse the adhered platelets. After 1 h at 37 °C, the releasing lactate dehydrogenase (LDH) enzyme was detected. The OD 490 value of the suspension was determined using a UV-visible spectrophotometer. The OD 490 nm value of the solution only containing 100 μL of PRP instead of material suspension was used as a reference value. The percentage of platelets adhered to the ointment was calculated using the following equation(2):

Percentage platelet adhesion (%platelet adhesion)

**= OD gauze / OD reference x 100%**

**Hemolysis assay**

Human blood obtained into EDTA tubes from volunteers was mixed with isotonic NaCl solution and centrifuged at 3000 RPM for 5 minutes. The cells that were packed were washed with isotonic saline and the cells were diluted to obtain a 5% red blood cell suspension in a 2 mL microcentrifuge tube. The ointment with varying concentrations was suspended using DI water. 0.5 mL of the ointment suspension along with 0.5 mL of the RBC suspension were incubated at 37°C for 1h in a microcentrifuge tube. After incubation, the tubes were centrifuged at 1000 rpm for 5 minutes. The supernatant was used to obtain absorbance readings at 560 nm. As a positive control, the sample was replaced by a 5%SDS solution, and the negative control contained an isotonic saline solution. The below equation was used to calculate the hemolytic activity(2).

**Hemolysis (%) =(A1-A2)/ (A3-A2)**

Al-absorbance of sample, A2-absorbance of negative control, A3-absorbance of positive control

**Clotting blood time**

10 mg of the sample was put into a test tube and preheated at 37 °C for 5 min. 1 mL of anticoagulant human blood from a healthy individual was added and incubated at 37 °C for 3 min. Then, 40 μL of a 0.2 mol/L CaCl2 solution will be added. The time was measured from the addition of CaCl2 until the blood clotting was designated as the clotting blood time. During this time, the blood was observed for clotting at 10s intervals. A blank group without adding samples was measured by the same procedure. At least three measurements in each group were taken(2).

References

1. Cheng Y, Yang Q, Wang J, Hu Z, Li C, Zhong S, et al. Optimization Preparation and Evaluation of Chitosan Grafted Norfloxacin as a Hemostatic Sponge. Polym 2023, Vol 15, [Internet]. 2023 Jan 28 [cited 2025 Dec 30];15(3). Available from: https://www.mdpi.com/2073-4360/15/3/672

2. Liu Y, Niu H, Wang C, Yang X, Li W, Zhang Y, et al. Bio-inspired, bio-degradable adenosine 5′-diphosphate-modified hyaluronic acid coordinated hydrophobic undecanal-modified chitosan for hemostasis and wound healing. Bioact Mater [Internet]. 2022 Nov 1 [cited 2025 Dec 31];17:162. Available from: https://pmc.ncbi.nlm.nih.gov/articles/PMC8965034/
