# Supplementary material for: Synergistic Bioactive Ointment: ZnO Nanoparticles Combined with Carica papaya Latex and Aloe Vera Gel for Broad-Spectrum Biomedical Applications
Source: PLoS One. 2026 Jul 21;21(7):e0353765. doi: 10.1371/journal.pone.0353765 (PMC13387519; doi:10.1371/journal.pone.0353765)

# Raw Data

| Whole blood coagulation assay (Blood clotting index) | | | | |  |
| --- | --- | --- | --- | --- | --- |
|  |  |  |  |  |  |
| **Trial 01** |  | **545 nm** | **800 nm** | **Reading** | **% BCI** |
| **Sample** |  |  |  |  |  |
| ***Control*** |  | 0.937 | 0.031 | 0.906 |  |
| Base |  | 0.016 | 0.002 | 0.014 | 1.545254 |
| ZnO |  | 0.153 | 0.05 | 0.103 | 11.36865 |
| CS |  | 0.027 | 0.006 | 0.021 | 2.317881 |
| PJ |  | 0.083 | 0.002 | 0.081 | 8.940397 |
|  |  |  |  |  |  |
| **Trial 01** |  |  |  |  |  |
| **Sample** |  |  |  |  |  |
| Base |  | 0.049 | 0.001 | 0.048 | 5.298013 |
| ZnO |  | 0.326 | 0.118 | 0.208 | 22.95806 |
| CS |  | 0.072 | 0.002 | 0.070 | 7.726269 |
| PJ |  | 0.093 | 0.003 | 0.090 | 9.933775 |
|  |  |  |  |  |  |
| **Trial 03** |  |  |  |  |  |
| **Sample** |  |  |  |  |  |
| Base |  | 0.036 | 0.001 | 0.035 | 3.863135 |
| ZnO |  | 0.411 | 0.117 | 0.294 | 32.45033 |
| CS |  | 0.15 | 0.021 | 0.129 | 14.23841 |
| PJ |  | 0.045 | 0.001 | 0.044 | 4.856512 |

| Erythrocyte adsorption/RBC attachment | | | | |  |  |
| --- | --- | --- | --- | --- | --- | --- |
|  |  |  |  |  |  |  |
| **Trial 01** |  | **540 nm** | **800 nm** | **Reading** |  |  |
| **Sample** |  |  |  |  |  |  |
| ***Control*** |  | 0.537 | 0.009 | 0.528 |  |  |
| Base |  | 0.244 | 0.2 | 0.044 |  |  |
| ZnO |  | 1.04 | 0.068 | 0.972 |  |  |
| CS |  | 0.803 | 0.058 | 0.745 |  |  |
| PJ |  | 0.199 | 0.085 | 0.114 |  |  |
|  |  |  |  |  |  |  |
|  |  |  |  |  |  |  |
| Platelet adhesion | | | | |  |  |
|  |  |  |  |  |  |  |
| **Trial 01** |  | **490 nm** | **800 nm** | **Reading** |  |  |
| **Sample** |  |  |  |  |  |  |
| ***Control*** |  | 0.457 | 0.036 | 0.421 |  |  |
| Base |  | 0.104 | 0.087 | 0.017 |  |  |
| ZnO |  | 0.164 | 0.121 | 0.043 |  |  |
| CS |  | 0.124 | 0.097 | 0.027 |  |  |
| PJ |  | 0.086 | 0.056 | 0.030 |  |  |
|  |  |  |  |  |  |  |
|  |  |  |  |  |  |  |
|  |  |  |  |  |  |  |

| Haemolysis | | | | |  |  |
| --- | --- | --- | --- | --- | --- | --- |
|  |  |  |  |  |  |  |
| **10 mg/mL** | | **560 nm** | **800 nm** | **Reading** | stability | % stability |
| **Sample** |  |  |  |  |  |  |
| ***Control*** |  | 2.97 | 0.152 | 2.818 |  |  |
| Base |  | 0.648 | 0.432 | 0.216 | 0.238411 | 23.84106 |
| ZnO |  | 0.243 | 0.15 | 0.093 | 0.102649 | 10.2649 |
| CS |  | 0.301 | 0.088 | 0.213 | 0.235099 | 23.50993 |
| PJ |  | 0.774 | 0.156 | 0.618 | 0.682119 | 68.21192 |
|  |  |  |  |  |  |  |
| **20 mg/mL** | |  |  |  |  |  |
| **Sample** |  |  |  |  |  |  |
| Base |  | 0.68 | 0.492 | 0.188 | 0.207506 | 20.75055 |
| ZnO |  | 0.352 | 0.191 | 0.161 | 0.177704 | 17.77042 |
| CS |  | 0.247 | 0.067 | 0.180 | 0.198675 | 19.86755 |
| PJ |  | 0.854 | 0.161 | 0.693 | 0.764901 | 76.49007 |
|  |  |  |  |  |  |  |
| **40 mg/mL** | |  |  |  |  |  |
| **Sample** |  |  |  |  |  |  |
| Base |  | 0.527 | 0.4 | 0.127 | 0.140177 | 14.01766 |
| ZnO |  | 0.359 | 0.082 | 0.277 | 0.30574 | 30.57395 |
| CS |  | 0.281 | 0.108 | 0.173 | 0.190949 | 19.09492 |
| PJ |  | 0.417 | 0.198 | 0.219 | 0.241722 | 24.17219 |

| **Sample** | **Trial 01** | **Trial 02** | **Trial 03** |
| --- | --- | --- | --- |
| ***Control*** | **4 min** | **5 min** | **6 min** |
| Base | 10 s | 20 s | 10 s |
| ZnO | 30 min | 16 min | 9 min |
| CS | 40 s | 60 s | 45 s |
| PJ | 10 s | 20 s | 10 s |

| ***Carica papaya* latex - DPPH** | |
| --- | --- |
|  |  |
| **Concentration** | **RSA %** |
| 100 | **10.75 ± 0.26** |
| 200 | **19.12 ± 0.51** |
| 300 | **31.25 ± 0.80** |
| 400 | **47.10 ± 3.20** |
| 500 | **57.50 ± 1.90** |
| 600 | **68.60 ± 2.00** |
| 700 | **78.62 ± 5.20** |
|  |  |
| ***Aloe barbadensis miller* gel - DPPH** | |
|  |  |
| **Concentration** | **RSA %** |
| 100 | **33.90 ± 0.44** |
| 200 | **45.42 ± 0.53** |
| 300 | **58.23 ± 0.52** |
| 400 | **69.90 ± 0.63** |
| 500 | **79.22 ± 0.45** |
| 600 | **88.06 ± 0.53** |
| 700 | **98.72 ± 0.56** |
|  |  |
| **ZnO nanoparticles - DPPH** | |
|  |  |
| **Concentration** | **RSA %** |
| 100 | **4.05917 ± 2.03** |
| 200 | **18.91985 ± 0.42** |
| 300 | **33.47093 ± 2.30** |
| 400 | **42.65566 ± 2.09** |
| 500 | **50.77399 ± 0.57** |
| 600 | **54.72996 ± 4.70** |
| 700 | **64.74028 ± 1.43** |

| ***Carica papaya* latex - Protease** | |
| --- | --- |
|  |  |
| **Concentration** | **Absorbance** |
| 1000 | **0.615** |
| 500 | **0.572** |
| 250 | **0.490** |
| 125 | **0.448** |
| 62.5 | **0.419** |
| 31.25 | **0.354** |
| 15.625 | **0.321** |
|  |  |
| **Tyrosine standard - Protease** | |
|  |  |
| **Concentration** | **Absorbance** |
| 500 | **0.747** |
| 400 | **0.685** |
| 300 | **0.616** |
| 200 | **0.511** |
| 100 | **0.432** |
| 50 | **0.366** |

| **Well diffusion method** | |  |  |  |  |  |
| --- | --- | --- | --- | --- | --- | --- |
|  |  |  |  |  |  |  |
| 10 mg/mL cream samples | | | (Base:Active ingredient = 70:30) | | |  |
|  |  |  |  |  |  |  |
| ***E.coli*** | **Inhibiton zone in mm** | | |  |  |  |
|  | **Plate 01** | **Plate 02** | **Plate 03** | **Average** | **SD** | **SE** |
| **Zno** | 13.50 | 12.00 | 12.00 | 12.50 | 0.87 | 0.50 |
| **Cream base** | 14.50 | 13.00 | 13.00 | 13.50 | 0.87 | 0.50 |
| **Papaya latex** | 12.00 | 12.00 | 12.00 | 12.00 | 0.00 | 0.00 |
| **Aloe gel** | 17.50 | 14.00 | 14.00 | 15.17 | 2.02 | 1.17 |
| **Pap:Aloe 1:1** | 13.00 | 12.00 | 12.00 | 12.33 | 0.58 | 0.33 |
| **Pap:Aloe 1:2** | 14.00 | 13.50 | 13.50 | 13.67 | 0.29 | 0.17 |
| **Pap:Aloe 2:1** | 13.00 | 13.00 | 14.00 | 13.33 | 0.58 | 0.33 |
| **Pap:Aloe 1:4** | 14.00 | 12.00 | 12.00 | 12.67 | 1.15 | 0.67 |
| **Pap:Aloe 4:1** | 13.50 | 12.50 | 12.50 | 12.83 | 0.58 | 0.33 |
|  |  |  |  |  |  |  |
| ***Staph*** | **Inhibiton zone in mm** | | |  |  |  |
|  | **Plate 01** | **Plate 02** | **Plate 03** | **Average** | **SD** | **SE** |
| **Zno** | 13.00 | 13.00 | 11.00 | 12.33 | 1.15 | 0.67 |
| **Cream base** | 13.50 | 13.00 | 12.00 | 12.83 | 0.76 | 0.44 |
| **Papaya latex** | 11.00 | 12.00 | 11.00 | 11.33 | 0.58 | 0.33 |
| **Aloe gel** | 11.00 | 12.00 | 11.00 | 11.33 | 0.58 | 0.33 |
| **Pap:Aloe 1:1** | 11.00 | 11.00 | 11.00 | 11.00 | 0.00 | 0.00 |
| **Pap:Aloe 1:2** | 10.00 | 11.00 | 12.00 | 11.00 | 1.00 | 0.58 |
| **Pap:Aloe 2:1** | 10.00 | 12.00 | 11.00 | 11.00 | 1.00 | 0.58 |
| **Pap:Aloe 1:4** | 10.50 | 12.00 | 12.00 | 11.50 | 0.87 | 0.50 |
| **Pap:Aloe 4:1** | 10.00 | 11.00 | 11.00 | 10.67 | 0.58 | 0.33 |

| 20 mg/mL cream samples | | | (Base:Active ingredient = 70:30) | | |  |
| --- | --- | --- | --- | --- | --- | --- |
|  |  |  |  |  |  |  |
| ***E.coli*** | **Inhibiton zone in mm** | | |  |  |  |
|  | **Plate 01** | **Plate 02** | **Plate 03** | **Average** | **SD** | **SE** |
| **Zno** | 15.50 | 15.00 | 15.00 | 15.17 | 0.29 | 0.17 |
| **Cream base** | 12.00 | 12.00 | 12.00 | 12.00 | 0.00 | 0.00 |
| **Papaya latex** | 11.00 | 11.00 | 11.00 | 11.00 | 0.00 | 0.00 |
| **Aloe gel** | 11.00 | 12.00 | 11.00 | 11.33 | 0.58 | 0.33 |
| **Pap:Aloe 1:1** | 12.50 | 12.50 | 13.50 | 12.83 | 0.58 | 0.33 |
| **Pap:Aloe 1:2** | 13.50 | 16.00 | 16.00 | 15.17 | 1.44 | 0.83 |
| **Pap:Aloe 2:1** | 17.50 | 17.50 | 15.00 | 16.67 | 1.44 | 0.83 |
| **Pap:Aloe 1:4** | 19.00 | 19.00 | 15.00 | 17.67 | 2.31 | 1.33 |
| **Pap:Aloe 4:1** | 16.50 | 16.50 | 15.50 | 16.17 | 0.58 | 0.33 |
|  |  |  |  |  |  |  |
| ***Staph*** | **Inhibiton zone in mm** | | |  |  |  |
|  | **Plate 01** | **Plate 02** | **Plate 03** | **Average** | **SD** | **SE** |
| **Zno** | 18.50 | 19.00 | 19.50 | 19.00 | 0.50 | 0.29 |
| **Cream base** | 13.00 | 13.50 | 13.00 | 13.17 | 0.29 | 0.17 |
| **Papaya latex** | 12.00 | 13.00 | 12.00 | 12.33 | 0.58 | 0.33 |
| **Aloe gel** | 12.00 | 12.00 | 12.50 | 12.17 | 0.29 | 0.17 |
| **Pap:Aloe 1:1** | 19.50 | 18.50 | 17.50 | 18.50 | 1.00 | 0.58 |
| **Pap:Aloe 1:2** | 20.00 | 17.50 | 18.00 | 18.50 | 1.32 | 0.76 |
| **Pap:Aloe 2:1** | 17.50 | 17.00 | 16.50 | 17.00 | 0.50 | 0.29 |
| **Pap:Aloe 1:4** | 21.00 | 20.00 | 20.00 | 20.33 | 0.58 | 0.33 |
| **Pap:Aloe 4:1** | 17.00 | 17.00 | 16.50 | 16.83 | 0.29 | 0.17 |

| 40 mg/mL cream samples | | | (Base:Active ingredient = 70:30) | | |  |
| --- | --- | --- | --- | --- | --- | --- |
|  |  |  |  |  |  |  |
| ***E.coli*** | **Inhibiton zone in mm** | | |  |  |  |
|  | **Plate 01** | **Plate 02** | **Plate 03** | **Average** | **SD** | **SE** |
| **Zno** | 15.50 | 18.00 | 14.50 | 16.00 | 1.80 | 1.04 |
| **Cream base** | 17.50 | 14.00 | 12.00 | 14.50 | 2.78 | 1.61 |
| **Papaya latex** | 14.00 | 13.00 | 13.50 | 13.50 | 0.50 | 0.29 |
| **Aloe gel** | 14.00 | 12.00 | 12.00 | 12.67 | 1.15 | 0.67 |
| **Pap:Aloe 1:1** | 12.50 | 12.50 | 12.00 | 12.33 | 0.29 | 0.17 |
| **Pap:Aloe 1:2** | 12.50 | 13.00 | 12.50 | 12.67 | 0.29 | 0.17 |
| **Pap:Aloe 2:1** | 12.00 | 13.50 | 13.00 | 12.83 | 0.76 | 0.44 |
| **Pap:Aloe 1:4** | 11.00 | 11.50 | 12.00 | 11.50 | 0.50 | 0.29 |
| **Pap:Aloe 4:1** | 11.00 | 11.00 | 10.50 | 10.83 | 0.29 | 0.17 |
|  |  |  |  |  |  |  |
| ***Staph*** | **Inhibiton zone in mm** | | |  |  |  |
|  | **Plate 01** | **Plate 02** | **Plate 03** | **Average** | **SD** | **SE** |
| **Zno** | 12.00 | 12.00 | 12.00 | 12.00 | 0.00 | 0.00 |
| **Cream base** | 12.00 | 12.00 | 12.00 | 12.00 | 0.00 | 0.00 |
| **Papaya latex** | 12.00 | 12.00 | 11.00 | 11.67 | 0.58 | 0.33 |
| **Aloe gel** | 11.00 | 11.00 | 10.50 | 10.83 | 0.29 | 0.17 |
| **Pap:Aloe 1:1** | 12.00 | 11.00 | 11.00 | 11.33 | 0.58 | 0.33 |
| **Pap:Aloe 1:2** | 10.00 | 11.00 | 10.50 | 10.50 | 0.50 | 0.29 |
| **Pap:Aloe 2:1** | 11.00 | 12.00 | 10.00 | 11.00 | 1.00 | 0.58 |
| **Pap:Aloe 1:4** | 10.50 | 11.00 | 11.00 | 10.83 | 0.29 | 0.17 |
| **Pap:Aloe 4:1** | 10.00 | 10.00 | 10.50 | 10.17 | 0.29 | 0.17 |

| 20 mg/mL cream samples | | | (Papain:Aloe = 1:4) | | |  |
| --- | --- | --- | --- | --- | --- | --- |
|  |  |  |  |  |  |  |
| ***E.coli*** | **Inhibiton zone in mm** | | |  |  |  |
|  | **Plate 01** | **Plate 02** | **Plate 03** | **Average** | **SD** | **SE** |
| **Base:Active ingredients 9:1** | 15.00 | 14.55 | 14.50 | 14.68 | 0.28 | 0.16 |
| **Base:Active ingredients 8:2** | 16.00 | 14.00 | 15.00 | 15.00 | 1.00 | 0.58 |
| **Base:Active ingredients 7:3** | 15.50 | 16.50 | 17.00 | 16.33 | 0.76 | 0.44 |
| **Base:Active ingredients 6:4** | 16.00 | 14.50 | 15.00 | 15.17 | 0.76 | 0.44 |
| **Base:Active ingredients 5:5** | 13.50 | 13.00 | 18.50 | 15.00 | 3.04 | 1.76 |
| **ZnO** | 14.00 | 15.50 | 16.50 | 15.33 | 1.26 | 0.73 |
| **Papaya latex** | 12.50 | 12.00 | 12.00 | 12.17 | 0.29 | 0.17 |
| **Aloe gel** | 10.50 | 14.50 | 12.50 | 12.50 | 2.00 | 1.15 |
|  |  |  |  |  |  |  |
| ***Staph*** | **Inhibiton zone in mm** | | |  |  |  |
|  | **Plate 01** | **Plate 02** | **Plate 03** | **Average** | **SD** | **SE** |
| **Base:Active ingredients 9:1** | 13.00 | 13.50 | 13.00 | 13.17 | 0.29 | 0.17 |
| **Base:Active ingredients 8:2** | 13.50 | 13.50 | 15.00 | 14.00 | 0.87 | 0.50 |
| **Base:Active ingredients 7:3** | 19.00 | 18.00 | 19.00 | 18.67 | 0.58 | 0.33 |
| **Base:Active ingredients 6:4** | 13.50 | 16.50 | 18.00 | 16.00 | 2.29 | 1.32 |
| **Base:Active ingredients 5:5** | 12.00 | 13.50 | 18.00 | 14.50 | 3.12 | 1.80 |
| **ZnO** | 17.50 | 15.00 | 14.00 | 15.50 | 1.80 | 1.04 |
| **Papaya latex** | 14.00 | 13.00 | 13.00 | 13.33 | 0.58 | 0.33 |
| **Aloe gel** | 14.00 | 12.50 | 12.50 | 13.00 | 0.87 | 0.50 |


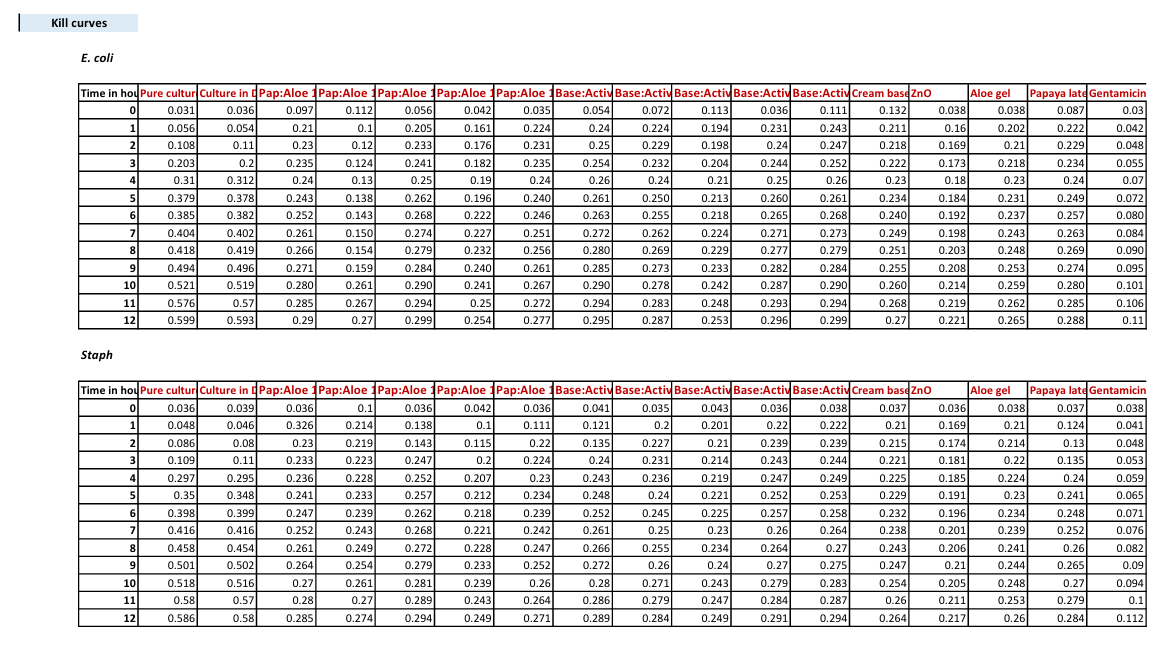

Supplement: S2 Section — All raw data from the experiments, including blood coagulation assay, RBC attachment, platelet adhesion, hemolysis, clotting blood time, DPPH assay, protease assay, agar well diffusion assay, and time kill curve data. (DOCX) [file pone.0353765.s002.docx]
