# Supplementary figures and images for: Synergistic Bioactive Ointment: ZnO Nanoparticles Combined with Carica papaya Latex and Aloe Vera Gel for Broad-Spectrum Biomedical Applications
Source: PLoS One. 2026 Jul 21;21(7):e0353765. doi: 10.1371/journal.pone.0353765 (PMC13387519; doi:10.1371/journal.pone.0353765)

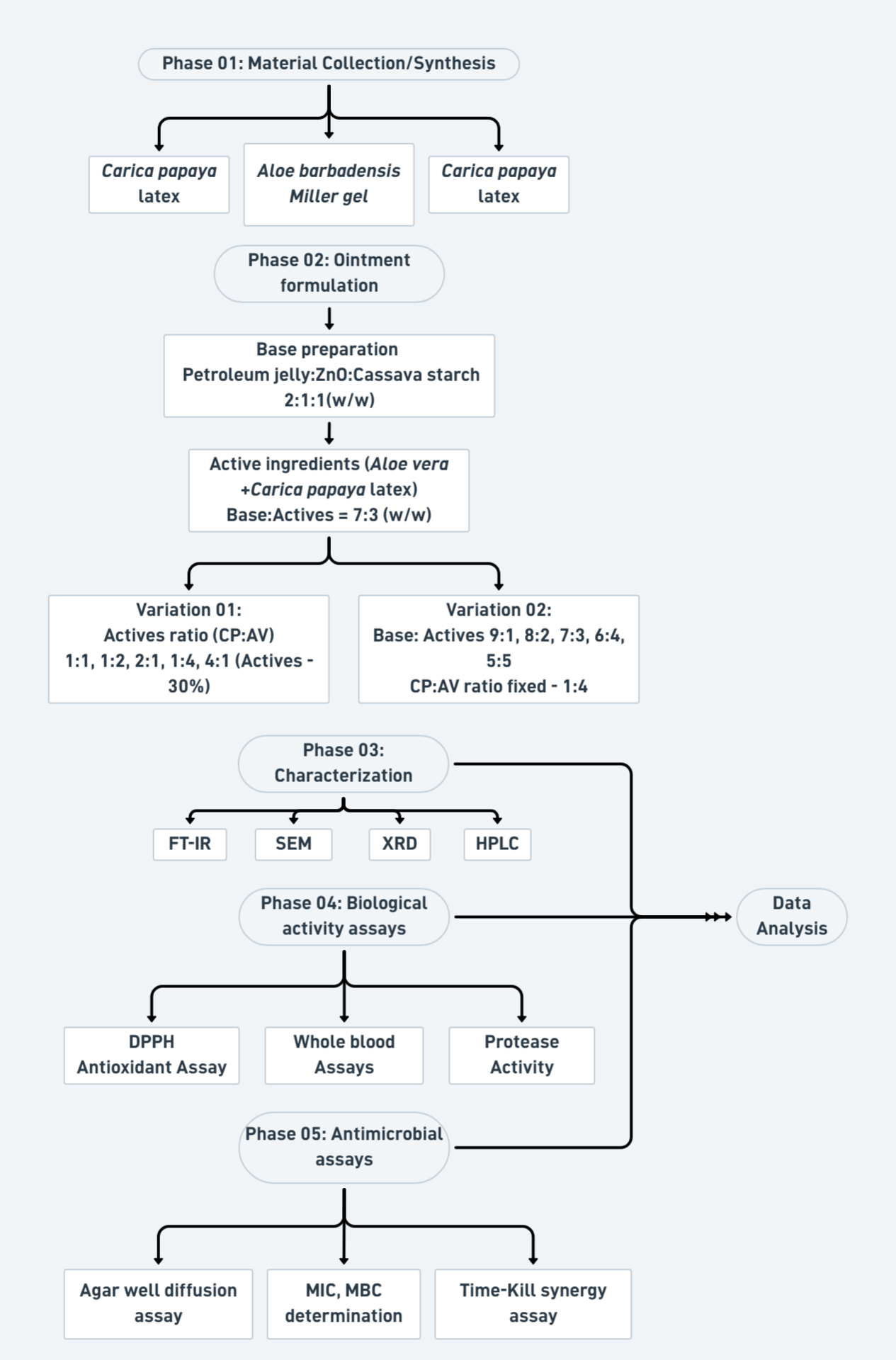

Supplement: S1 Scheme — (TIF) [file pone.0353765.s003.tif]

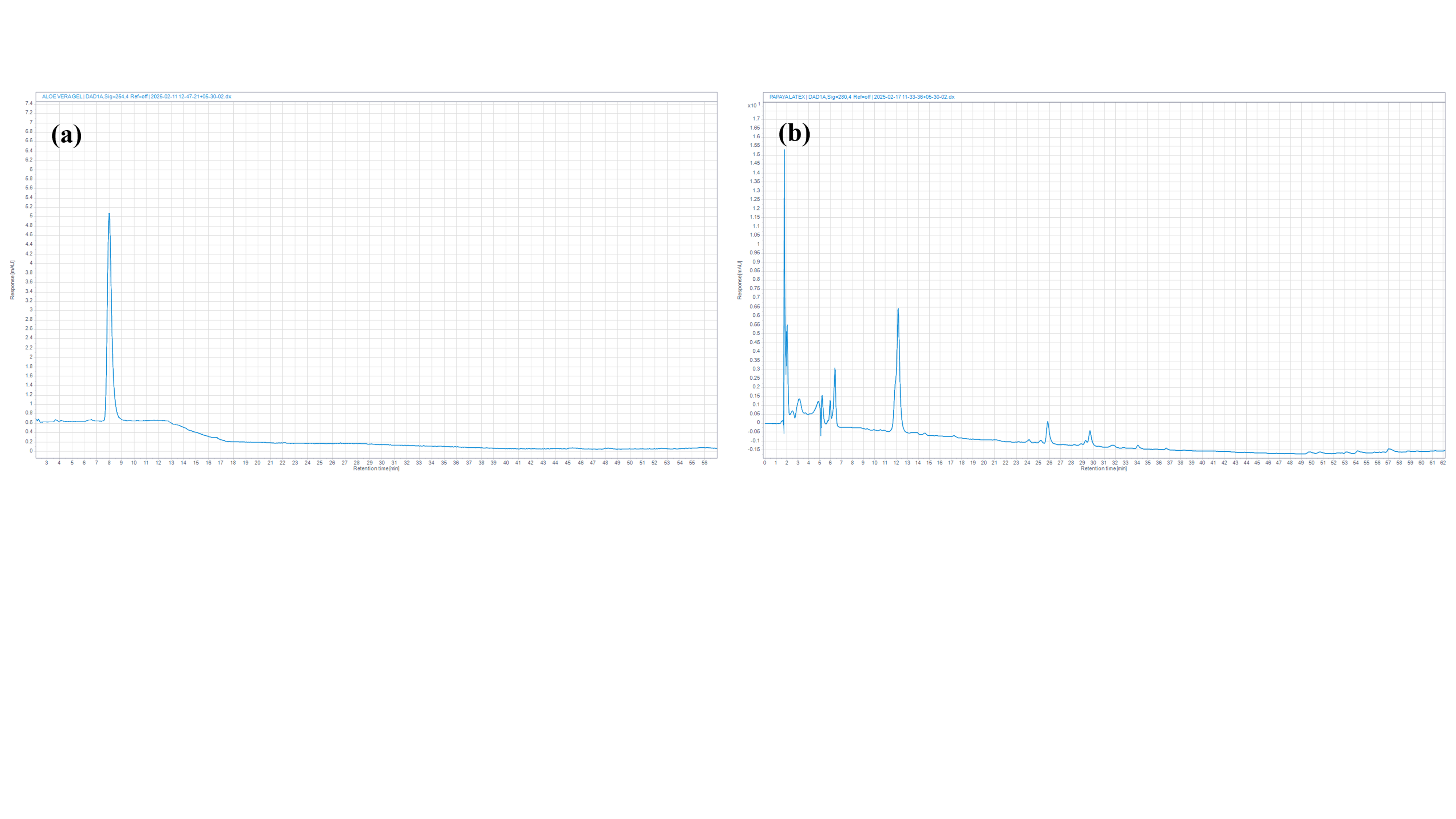

Supplement: S1 Fig — (TIF) [file pone.0353765.s004.tif]

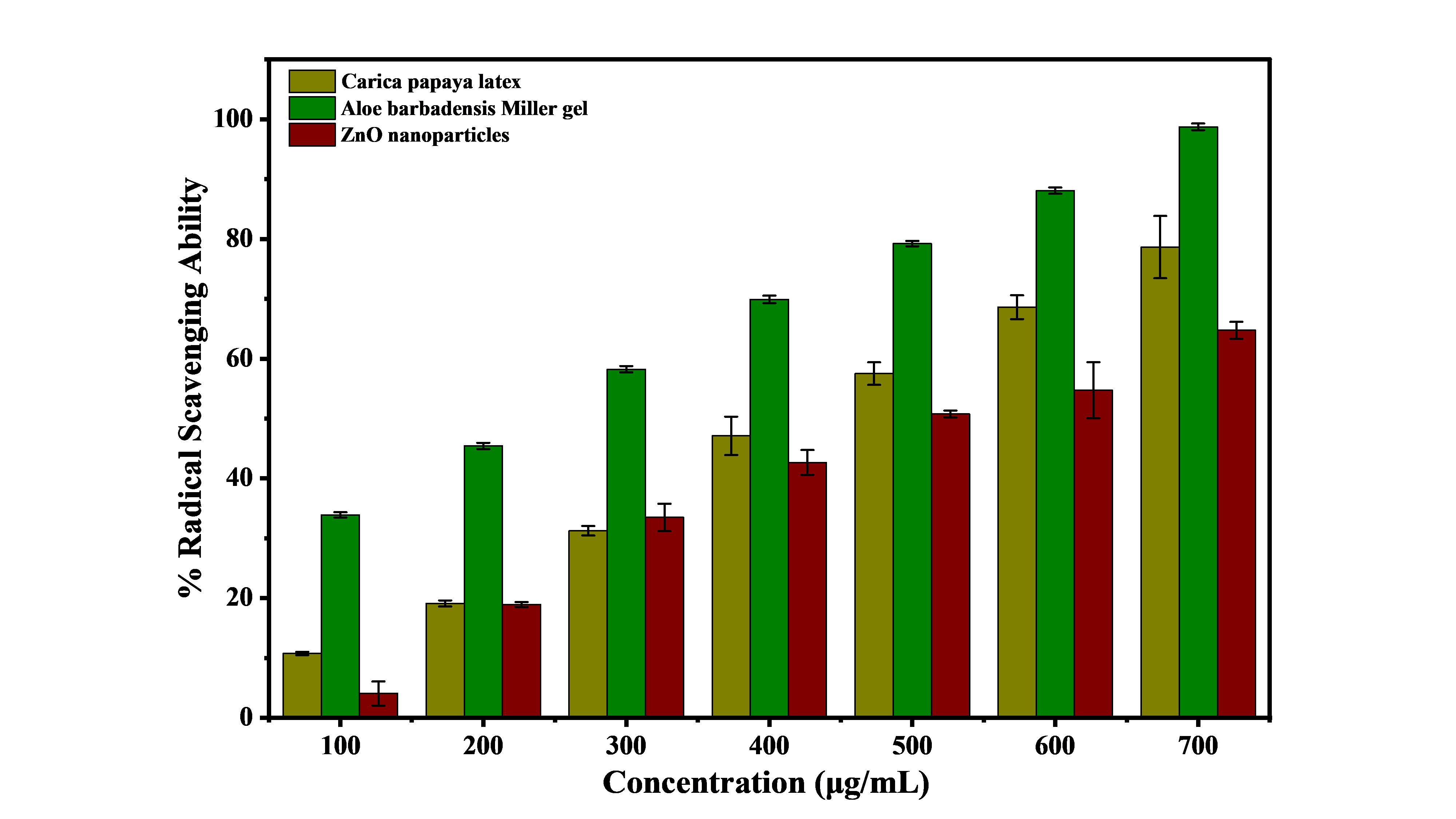

Supplement: S2 Fig — (TIF) [file pone.0353765.s005.tif]

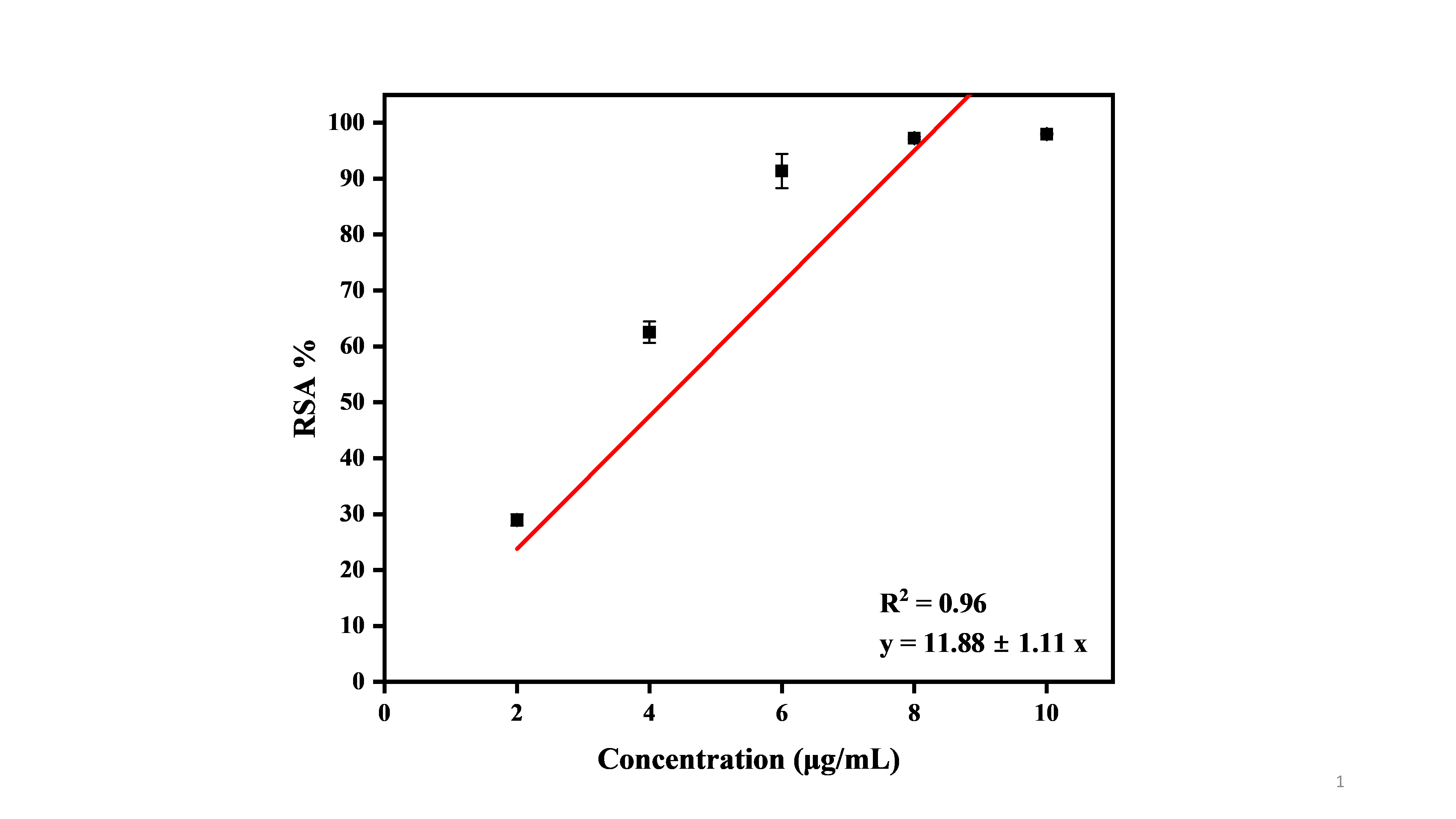

Supplement: S3 Fig — (TIF) [file pone.0353765.s006.tif]

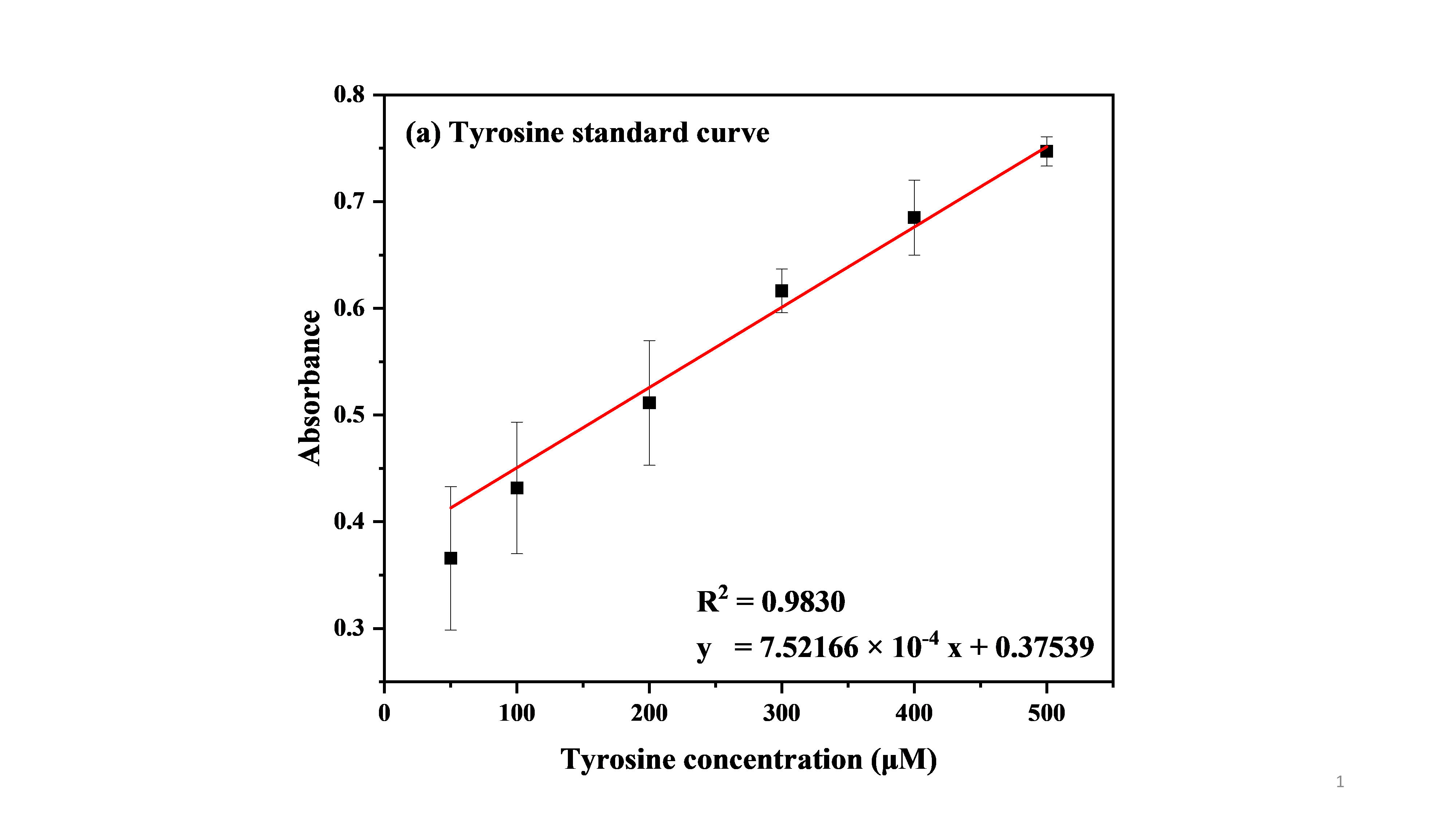

Supplement: S4 Fig — (TIF) [file pone.0353765.s007.tif]
